# A functional two-pore-domain K^+^ channel from a large DNA virus

**DOI:** 10.1073/pnas.2626158123

**Published:** 2026-10-23

**Authors:** Oliver Rauh, Sebastian Mach, Oliver Clarke, Jonas Toews, James L. Van Etten, Mike Althaus, Anna Moroni, Gerhard Thiel

**Affiliations:** ^a^ Institute for Functional Gene Analytics, Department of Natural Sciences, Bonn-Rhein-Sieg University of Applied Sciences, Rheinbach 53359, Germany; ^b^ Department of Biology, Membrane Biophysics, Technische Universität Darmstadt, Darmstadt 64287, Germany; ^c^ Department of Anesthesiology, Columbia University Irving Medical Center, New York, NY 10032; ^d^ Department of Biology, Irving Institute for Clinical and Translational Research, Columbia University, New York, NY 10032; ^e^Department of Plant Pathology and Nebraska Center for Virology, University of Nebraska Lincoln, Lincoln, NE 68583-0900; ^f^ Department of Biosciences, University of Milan, Milan 20133, Italy

**Keywords:** giant viruses, 2 pore potassium ion channels, evolution

## Abstract

The evolutionary history of proteins helps to understand their structure/function correlates and suggests on how functional complexity evolved from primitive precursors. In the superfamily of K^+^ channels, functional features of complex eukaryotic inward rectifier (Kir) channels and voltage-dependent (Kv) channels could be understood in terms of an increase in structural complexity of their prokaryotic precursors. K2P channels, which are made from tandem repeats of two pore domains, are not found in prokaryotes and their evolutionary origin is still unknown. In searching for primitive K2P channel precursors, we screened the genomes of nucleocytoplasmic large DNA viruses (NCLDVs) for genes coding for K2P channels. This was motivated by two reasons: NCLDVs are already known for coding primitive Kir type channels, and they play important roles in the evolution of eukaryotes. Here, we report that the NCLDV *Pyramimonas orientalis* virus codes for K2P-like K^+^ channels. With only 178 amino acids and no cytosolic domains, this Kpov protein is much smaller than any eukaryotic K2P channel, but still functional as K^+^ selective channel in lipid bilayers. Phylogenetic analysis suggests that Kpov is not the product of molecular piracy from the host but may result from fusion of primitive viral K^+^ channel pore domains.

## Significance

While the evolutionary history of the pore and its sensor domains in modern K^+^ channels can be traced back to prokaryotes, the origin of a tandem architecture remains unknown; such structures do not occur in bacteria. Here, we highlight a possible contribution by giant dsDNA viruses to K^+^ channel evolution. A member of the giant Mimiviruses, which infects a single-celled marine alga, contains a gene for a tandem K^+^ channel with structural hallmarks of K^+^ channel pores. This channel is functional and exhibits a 10-fold selectivity for K^+^ over Na^+^. As eukaryotic cells have acquired genes from DNA viruses during evolution, these viruses may have acted as mediators of horizontal gene transfer or as primary source of K^+^ channel genes.

**Fig. 1.**
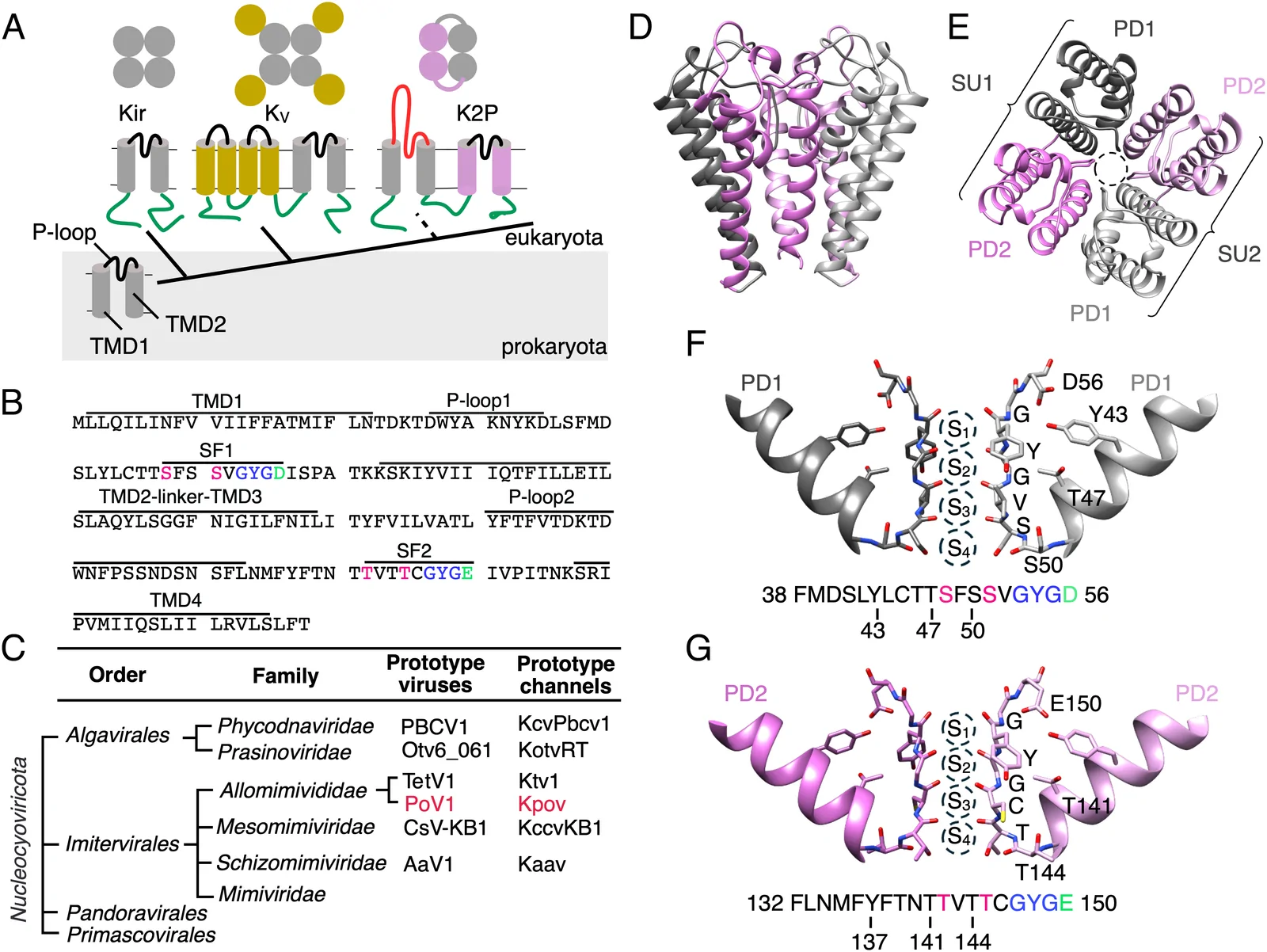
Modular evolution of K^+^ channels and architecture of viral K2P-like channel Kpov. (*A*) K^+^ channels presumably evolved from basic pore-forming monomers with a conserved P-loop domain which contains the selectivity filter and connects two transmembrane domains TMD1 and TMD2. During evolution the pore module acquired additional building blocks with sensor function as cytosolic domains (green) and/or as additional TMDs (yellow). This led to distinct K^+^ channel families with diverse structural and functional features such as inward rectifiers (Kir) and voltage gated channels (Kv). A specific case is Two-Pore channels (K2P) where the functional K2P channel is a dimer of two subunits (gray and pink), each subunit containing two pore domains and four pore domains forming the functional pore. Top row: Kir and Kv subunits assemble in a functional channel as a tetramer where the pore module forms the central unit. In K2P channels the functional protein is made of dimers of monomers with two pore domains. A structural peculiarity of animal K2P channels is an extended loop in the first pore domain (red), which forms the typical helical cap at the entrance to these channels. K^+^ channels with a Kir and Kv type architecture were discovered in prokaryotes underpinning that they already occurred early in evolution. K2P type channels have not been discovered in prokaryotes. The dotted line indicates one possible hypothesis according to which these channels may have first occurred in early extinct eukaryotes. (*B*) Primary sequence of Kpov. Position of transmembrane helices (TMD) 1 to 4, pore helices (P-loop), and selectivity filter domains (SF) are underlined; critical amino acids of the K^+^ channel signature sequence of the two SFs are indicated in color: the GYG motif in blue, and the position of a canonical threonine upstream (red) and anionic amino acid downstream of GYG (green). (*C*) Simplified phylogeny of *Nucleocytoviricota* according to (9) including prototype viral K^+^ channels and viruses in which the respective genes were detected. The Kpov channel originates from PoV, in the family of *Allomimiviridae*. (*D*) Side view and (*E*) top view of Kpov channel structure as predicted by AlphaFold2 based on the amino acid sequence shown in *B*. The two pore domains (PD1 and PD2) are colored in gray and pink, respectively, and the position of the individual pore domains in the tandem organization are indicated. (*F* and *G*) Side-view orientation with two opposing monomers of PD1 (*F*) and PD2 (*G*) with indication of structural details and corresponding selectivity filter sequences in which the consensus motif is color-coded as in *B*. The dashed circles in *B*, *F*, and *G* mark the position of the ion pathway with location of K^+^ binding sites S_1_–S_4_.

Potassium channels are membrane proteins which catalyze the selective diffusion of K^+^ ions across membranes of all organisms. A central building element, common to all K^+^ channel proteins, is the pore module in which four monomers group around a central water-filled pore serving as an ion conducting pathway (1). The tetramer-forming monomers are typically made of two transmembrane domains (TMD1 and TMD2) connected via a loop, the P-loop (2, 3) (Fig. 1*A*). The latter contains the canonical K^+^ channel consensus sequence TxxTxGY/FG, which forms the selectivity filter (4). In eukaryotic K^+^ channels this highly conserved pore module domain is fused to several different transmembrane and/or cytosolic domains. This results in a variety of K^+^ channel families with distinct structural and functional properties. The most common among these K^+^ channel families are the inward rectifiers (Kir), the voltage-gated channels (Kv), and the two-pore-domain channels (K2P) (Fig. 1*A*). The latter differ from the conserved scheme of the tetrameric K^+^ channel architecture in that the pore-forming subunits are organized in tandem pairs of two K^+^ channel sequences (Fig. 1*A*). A dimer of such a tandem pair forms the functional K2P channel (5–7). K^+^ channels with such a tandem pore architecture are found in algae and vascular plants as well as in lower and higher animals (8). A specific structural hallmark of K2P channels from animals is an extended loop structure in the first pore domain (Fig. 1*A*), which forms a so-called helical cap at the extracellular entrance of these channels (5).

A bulk of bioinformatic and experimental evidence supports the view that structural and functional diversity of K^+^ channels evolved in a modular manner (1, 10–12). A likely origin was a primordial channel pore in the procaryotic world, which resembles the conserved tetrameric pore module of all K^+^ channels (13). This central building block already contains the structural elements for the most basic functional features of K^+^ channels, namely the selectivity filter for discriminating between K^+^ and Na^+^ ions as well as the channel gates (2). During evolution, this pore module presumably acquired large cytosolic domains and/or additional peripheral transmembrane domains with sensory functions (2, 14). (Fig. 1*A*). In this way channels gained the possibility to sense diverse chemical and physical cues and translate them into a regulated opening and closing of the gates in the pore module. In modern Kir channels, for example, the basic pore module is connected to extended cytosolic domains with ligand binding sites (15, 16), which control channel activity. Another example is found in the family of Kv channels which presumably evolved from a fusion of the pore module to a voltage sensor domain (1, 10, 11) (Fig. 1*A*). The latter is built of four TMDs with a distinct pattern of charged amino acids, providing the mechanistic basis for control of the gates in the pore module by membrane voltage.

The discovery of structural homologs of eukaryotic Kir- channels and Kv- channels in bacteria and archaea suggested that their modular evolution and the associated functional diversification has already occurred in a prokaryotic world (17–22) (Fig. 1*A*). The evolutionary origin of K2P channels is less clear (5) (Fig. 1*A*). Unlike Kv-type and Kir-type channels, K2P channels have so far not been detected in genomes of procaryotes, which makes it more difficult to trace their evolutionary history (23, 24). Currently, there are two hypotheses on their origin: One hypothesis suggests that they originated from a gene duplication, which may have occurred in early extinct eukaryotes (8) (Fig. 1*A*). The second hypothesis suggests that they emerged from a fusion of two different genes in prokaryotes and that these prokaryotic ancestors no longer exist (23).

Most considerations on channel evolution ignore the contribution of viruses in shaping eukaryotic genomes. Recent studies however provide several arguments why this may be short sighted. One reason for considering also a role of viruses in the evolution of K^+^ channels is that genes, which code for potassium channels are indeed present in some nucleocytoplasmic large DNA viruses (NCLDV) (25–27). From a structural perspective, these Kcv-type (for K^+^ channel from Chlorella virus) channels and its relatives are minimal in size and structurally reduced to not more than the aforementioned pore module of all K^+^ channels (Fig. 1*A*) (26, 28). Despite their small size, they still exhibit the canonical function of K^+^ channels including a high K^+^/Na^+^ selectivity and sensitivity to typical K^+^ channel blockers such as Ba^2+^ and TEA^+^ (29). Like most other K^+^ channels they also possess two gates, the filter gate and the inner gate at the entry to the cavity (28, 30).

A second reason why viruses may have played a role in K^+^ channel evolution is supported by increasing evidence for gene transfer from viruses to their host. A systematic analysis has demonstrated that many eukaryotic lineages have indeed acquired a diverse range of genes from DNA viruses throughout their evolutionary history (31). In the context of K^+^ channel evolution it is interesting to mention that this directional gene transfer was observed for double-stranded DNA viruses and in particular for NCLDVs, including *Phycodnaviridae* and *Mimiviridae* (31). Notably both types of viruses are a rich source of genes for the aforementioned miniature sized viral K^+^ channels (25–27, 32). A phylogenetic analysis of these viral K^+^ channels has shown that they are not related to the K^+^ channels of their host (33, 34); hence, they are not the result of molecular piracy from the host, but they may have rather served as a source for an early host.

In case eukaryotic cells have acquired K^+^ channel genes from large DNA viruses during evolution, the latter may have either served as a shuttle for horizontal gene transfer or even as the primary source of channel genes (31). Such a contribution of viruses to the genetic exchange across eukaryotic diversity is very plausible considering that many algae exhibit an endogenization of large DNA viruses. This viral DNA contains, among other genes, also genes for ion channels (35). The viruses, which infect the respective algae namely *Chlorella*, *Micromonas,* and *Ostreococcus* all contain genes encoding K^+^ channels (25, 26, 32).

The hypothesis that viruses may have played an important role in K^+^ channel evolution is also relevant for an understanding of the origin of K2P channels. Bioinformatic analysis of plant K2P channels (7) concluded that plants may have acquired one of the pore domains by virus mediated horizontal gene transfer. The finding that ion channel homologues of these pore modules are also present, as mentioned before, in the large DNA viruses like Chloroviruses and Prasinoviruses lends further support to this hypothesis (7).

The unknown origin of K2P channels as well as the circumstantial evidence for a role of viruses in the early evolution of K^+^ channels raise the question whether primitive K2P type channels exist in viral genomes. To answer this question, we searched the protein data bank of viral genomes for potential K^+^ channel proteins with a tandem architecture. We identified a gene in the giant *Pyramimonas orientalis* virus (PoV1), a member of the NCLDV infecting a flagellated unicellular green alga (36), which codes for a 178 amino acid long protein with the structural hallmarks of a K2P channel. With functional reconstitution of this protein in planar lipid bilayers, we show that the protein generates an active K^+^ channel with a distinct selectivity for K^+^ over Na^+^. A phylogenetic analysis suggests that the viral K2P channel is not the result of recent molecular piracy from a host; the data are more compatible with the idea that the tandem pores are of a viral origin.

## Results and Discussion

In search for viral K2P type channels, we screened the National Center for Biotechnology Information (NCBI) data bank of viral genomes for proteins with two K^+^ channel filter motifs linked in tandem and found a candidate protein (Fig. 1*B*) (Protein data bank identity code QOI90491.1, *SI Appendix*, Table S1) in the *P. orientalis* virus (PoV1), a marine virus isolated from Norwegian coastal waters. With its large dsDNA genome of 560 kpb, it belongs to the Phylum *Nucleocytoviricota* (Fig. 1*C*); the unicellular green alga *P. orientalis* serves as the host (36). It is interesting to note that this same virus as well as its host were previously found as a source of unusual anion conducting Channelrhodopsins (37).

**Fig. 2.**
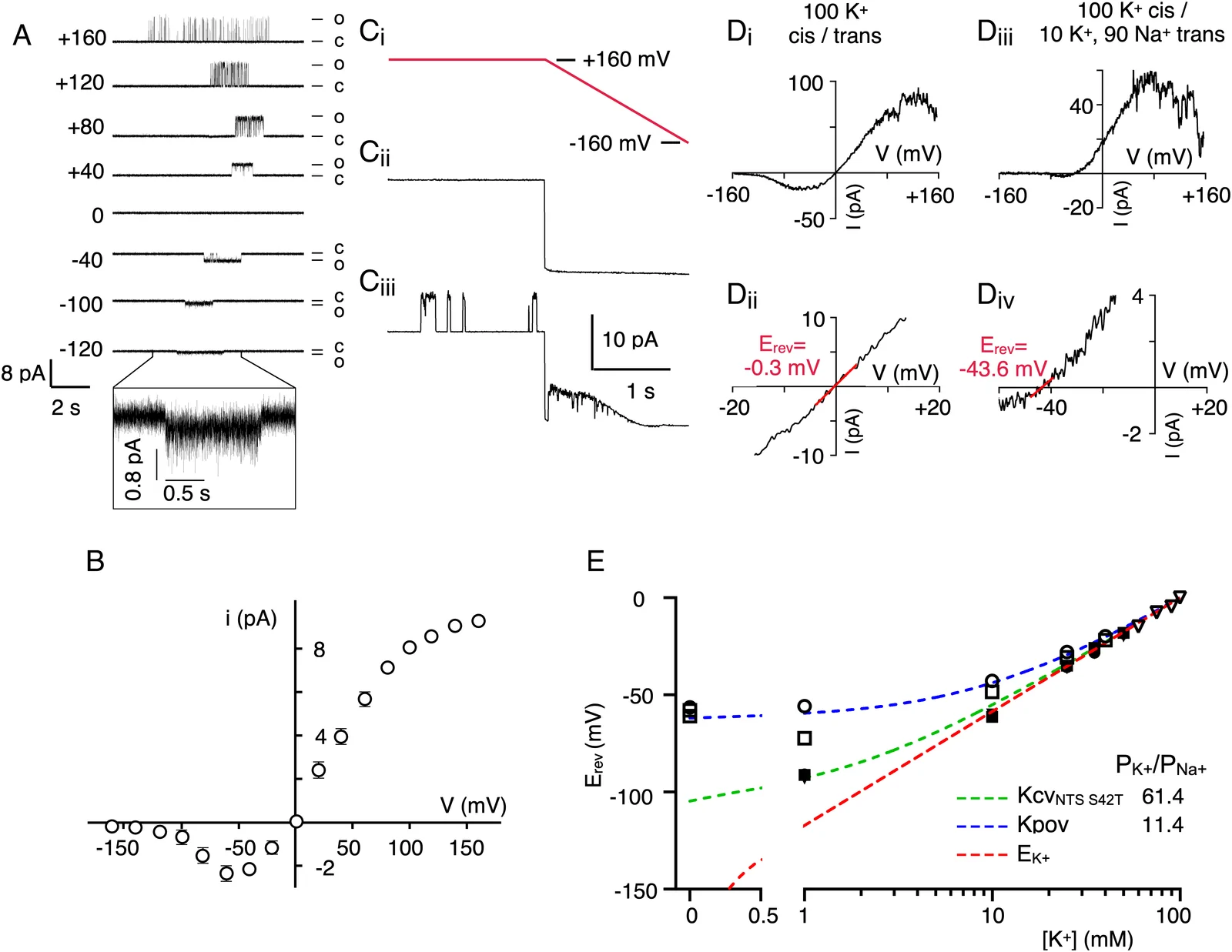
Functional properties of Kpov. (*A*) Representative single channel current traces of Kpov in DPhPC bilayers at voltages given on the left. Open (o) and closed (c) levels are marked alongside traces. Inset: magnification of flicker type channel opening from time series at −120 mV. (*B*) Corresponding mean i/V-relationship (±SD, N = 4) of Kpov in symmetrical 100 mM KCl (open circles). For estimating reversal voltage (E_rev_), bilayers with functional Kpov channels were repetitively challenged by voltage protocols comprising a 5 to 10 s step to holding voltage (+160 mV) followed by a ramp (160 mV/s) to −160 mV (*C_i_*). Current responses to the voltage protocol in *C_i_* with the channel being closed (*C_ii_*) or open (*C_iii_*) (here in 100 mM K^+^ in *cis* and 10 mM K^+^ + 90 mM Na^+^ in *trans*). Macroscopic open channel I/V relation in symmetrical 100 mM KCl (*D_i_* and *D_ii_*) or asymmetrical 100 mM K^+^ in *cis* and 10 mM K^+^ + 90 mM Na^+^ in *trans* (*D_iii_* and *D_iv_*). Macroscopic open channel currents were obtained by subtracting traces without channel activity during the voltage ramp from currents with open channels. Mean I/V relationship of open channel from the same bilayer in different ionic conditions over the entire voltage span between ±160 mV (*D_i_* and *D_iii_*) and magnification of the traces around the reversal voltage (*D_ii_* and *D_iv_*). E_rev_ was obtained from a linear fit of data close to reversion (red lines in *D_ii_* and *D_iv_*). (*E*) E_rev_ values of Kpov (open symbols) and Kcv_NTS S42T_ (filled symbols) obtained as in *D* as a function of variable K^+^/Na^+^ concentrations. In three independent experiments for each channel, one chamber initially contained 100 mM KCl and the other 100 mM NaCl. Na^+^ was then gradually replaced by K^+^ during the experiment, keeping the total cation concentration constant. The red line illustrates expected E_rev_ values as a function of K^+^ concentration for a perfectly selective K^+^ channel according to the Nernst equation; E_rev_ values for Kpov (dashed blue line) and Kcv_NTS S42T_ (dashed green line) were fitted with the GHK constant field equation. Best joint fits yielded P_K+_/P_Na+_ values of 11.4 (95% CI: 9.7 to 13.5) and 61.4 (95% CI: 50.1 to 76.6) for Kpov and Kcv_NTS S42T_, respectively.”

The derived amino acid sequence of a monomer of the putative viral K2P channel Kpov (for K^+^ channel from *P. orientalis* virus; for further information on nomenclature see *SI Appendix*, Table S1) as well as the AlphaFold2 predicted monomeric and dimeric structures are shown in Fig. 1 *D*–*G*. The sequence of the monomer contains two copies of the K^+^ channel filter sequence with the conserved GYG motif of K^+^ channels (Fig. 1 *B*, *F*, and *G*). Each of these consensus motifs is part of a typical K^+^ channel pore module where the filter sequence is positioned between two TMDs and downstream of a short pore helix (Fig. 1 *F* and *G*). With this global architecture the viral protein resembles the general structure of K2P channels [Fig. 1*A*; (2, 5)]. Two of these monomers assemble in a dimer in a way that the selectivity filter sequence forms a water filled pore for the transport of ions. The general arrangement of the two monomers in the functional channel unit is shown in the predicted structure in Fig. 1 *D*–*H* in a side view and in a top view. In the latter perspective it is seen how the selectivity filter loops are forming the pore for K^+^ permeation.

The fact that both selectivity filter sequences contain the GYG motif is more typical of plant tandem channels; in animal K2P channels the second filter motif frequently contains a Phe (F) or a Leu (L) instead of a Tyr (Y) (38). The presumed relationship of the viral protein to plant K2Ps is also supported by two other features: First, like in plant K2P channels the GYG motif in the viral protein is in both pore domains followed by an anionic amino acid; in animal K2Ps this position in the first pore domain is mostly occupied by a neutral amino acid (38). Second, an even more pronounced structural similarity between the viral K2P channel and plant homologs is that both lack the outer cap domain in the first pore domain (Fig. 1 *B* and *D*–*H*), which is typical of animal K2P channels (5) (Fig. 1*A*).

Further inspection of the filter motifs reveals that the TVTTCGYG sequence in the second pore domain of Kpov is in perfect agreement with the consensus filter sequence of all K^+^ channels (TXXTXGYG) (Fig. 1*G*). The motif in the first pore domain however is more unusual in that both consensus Thr are substituted by a Ser (Fig. 1*F*). While a Ser in the first position is also found in other channels like Kv1.1 or TWIK-1 (38) a Ser in the second position seems to be rarer. If we use the filter sequence of the first pore domain (SFSSVGYG) as a query, a BLAST search of non-K2P channels actually reveals only a few nonviral K^+^ channels that contain a -SXGYG- motif. One (SFASVGYG) for example was identified in the multicellular green alga *Klebsormidium nitens* (Gene bank nr: GAQ78956.1) another (SSFSIGYG) in putative channels from different *Leishmania* species (e.g., Gene bank nr: KAL0515581.1). Many of the K^+^ channel proteins from marine viruses, including some with a confirmed channel function, on the other hand, contain a Ser in this position (26). Worth mentioning here is the filter motif TTMSSVGYG in Kotu_6_1 (*SI Appendix*, Table S1), which is nearly identical to the noncanonical filter sequence in Kpov (Fig. 1*F*).

### Kpov Is a Functional K^+^ Selective Ion Channel.

Since annotation of a viral protein as a K^+^ channel is not a guarantee of its function (27) we expressed the protein in vitro into nanodiscs and reconstituted it in planar lipid bilayers (39). This generated robust channel fluctuations (Fig. 2*A*) confirming that the Kpov protein is indeed an ion channel. The channel exhibits a low open probability (Po ~ 0.01 to 0.05) with well resolved openings and closings at voltages >−40 mV. This gives rise to a near linear i/V relationship in a voltage window between −40 mV and +80 mV with an open channel conductance of 86 ± 1.2 pS. At voltages negative to −40 mV the Kpov channel shows an increase in open channel noise. This phenomenon is typical for most K^+^ channels of viral origin and reflects a fast voltage-dependent gating in the selectivity filter (30, 40). With increasing hyperpolarization this type of filter gating becomes progressively faster, with the effect that the full channel opening is no longer resolved by the recording system. As a result, the apparent open channel current decreases with increasing hyperpolarization. This generates an apparent i/V relation with a negative slope conductance at hyperpolarizing voltages (Fig. 2*B*), which is typical for most viral K^+^ channels (28, 40, 41). In analogy to other Kcv channels with the same fast gating and with a defined orientation in the bilayer (39, 42, 43), we can assign negative currents of Kpov in the bilayer recordings as inward currents.

To test the ion selectivity of Kpov, we employed a combined voltage-step/voltage-ramp protocol (Fig. 2*C*) in which a bilayer with active Kpov channels was first stepped for 5 to 10 s to +160 mV to activate the channels. The voltage was then changed by a fast ramp (160 mV/s) to −160 mV. Fig. 2*C* shows the current responses to two exemplary ramps, one without (Fig. 2*C_ii_*) and one with an open channel during the voltage ramp (Fig. 2*C_iii_*). To eliminate the capacitive current component the mean current of ≥10 ramps without an active channel was subtracted from all current traces with active channels during the ramp. All background corrected traces were then summed to derive the open channel I/V relationship. An example for such an open channel I/V relation recorded in symmetrical 100 mM KCl is plotted in Fig. 2*D_i_*. In a magnification of the data (Fig. 2*D_ii_*) the currents close to the point of current reversion can be fitted with a linear function providing a reversal voltage at −0.3 mV, e.g., a value close to the expected 0 mV. In a second set of experiments the KCl solution in the *trans* chamber was initially replaced by 100 mM NaCl. The Na^+^ concentration in the *trans* chamber was subsequently stepwise replaced by K^+^ giving final K^+^ concentrations of 1 mM, 10 mM, 25 mM, and 40 mM. The exemplary background corrected open channel I/V relationship with 10 mM K^+^ and 90 mM Na^+^ in the *trans* chamber shows a left shift of E_rev_ to −43.6 mV consistent with a K^+^ selectivity of Kpov (Fig. 2 *D_iii_* and *D_iv_*). In a plot of the measured reversal voltages as a function of the K^+^ concentration on the *trans* side (Fig. 2*E*) the E_rev_ value progressively deviates with decreasing K^+^ concentration from the theoretically expected Nernst voltage of an ideally selective K^+^ channel. This suggests that Kpov conducts to some extent also Na^+^. A joint fit of the data from three independent experiments with the Goldman–Hodgkin–Katz (GHK) equation yields a permeability ratio of P_K+_/P_Na+_ of 11.4 [95% confidence interval (CI): 9.7 to 13.5]. Notably, the fit extrapolates well to E_rev_ values recorded in buffers with a transmembrane gradient of 100 mM K^+^/100 mM Na^+^ (Fig. 2*E*).

To test if this relatively low P_K+_/P_Na+_ value is related to the method for analyzing the selectivity, we repeated the same experimental procedure with the Kcv_NTS S42T_ channel. The latter is a K^+^ selective Kcv type channel with the canonical tetrameric assembly of four monomers, each containing the consensus filter sequence (**T**HT**T**V**GYG**) typical for highly selective K^+^ channels (28). In these experiments we obtained with the same procedure for three independent recordings a P_K+_/P_Na+_ value of 64.4 (Fig. 1*E*). This higher preference for transport of K^+^ over Na^+^ is in good agreement with the well-known selectivity of these channels. Taken together, the data suggest that Kpov is a K^+^ channel but with a moderate selectivity for K^+^ over Na^+^.

The reported P_K+_/P_Na+_ ratios from K2P channels are in the order of 40 to 500 (44–47) and hence in the same order of those reported for tetrameric K^+^ channels (48). This implies that the asymmetry of the Kpov filter is not per se a reason for its lower selectivity. However, it is also known that the inherent asymmetric filter architecture in K2P channels makes this domain more flexible than that of canonical K^+^ channels. Consequently, mutations outside of the filter sequence can result in a significant lowering of their ability to discriminate between K^+^ and Na^+^ (49). An interesting example was found in *C. elegans* where a K2P-type channel exhibits a peculiarly high Na^+^ permeability (50). In this case, the low selectivity was causally related to a Cys in the first pore just upstream of the GYG motif. Interestingly, the Kpov channel contains the same CGYG motif in the second pore domain (Fig. 1 *F* and *G*). It is reasonable to speculate that this sequence specificity in Kpov might be the reason for its low P_K+_/P_Na+_ ratio.

### Kpov Is Not the Result of Recent Molecular Piracy from the Virus Host.

To compare the tandem viral channel Kpov with K2P channels from other organisms, its sequence was blasted against the NCBI data bank of nonredundant protein sequences. The two most similar proteins (Khf, Kdt) (listed in *SI Appendix*, Table S1) are annotated, as expected from the predicted structure, as K2P channels. *SI Appendix*, Fig. S1*C* shows an alignment of the Kpov sequence with the most similar Khf protein from the slime net *Hondaea fermentalginana*. Both proteins share the typical K^+^ channel signature sequences in tandem. However, the overall identity between the proteins is only 26.5%. The viral protein is only 178 amino acids per monomer, which is also much smaller than all nonviral orthologs (*SI Appendix*, Table S1). Thereby the Kpov protein follows the general trend of Kcv-type channels, which turn out to be generally much smaller than their pro- or eukaryotic orthologs (51). From the predicted structure (Fig. 1 *D*–*F*) and from a direct comparison to the Khf protein, it becomes apparent that the viral protein lacks any appreciable cytosolic N and C termini. Hence, it lacks critical structural elements, which are important for the gating of plant and animal K2P channels (52, 53). Yet, our functional data confirm that Kpov is still able to gate; it exhibits the characteristic voltage-dependent flicker gating of Kcv-type channels (Fig. 2 *A* and *B*), which is caused by a distinct type of filter gate in these channels (28).

To further understand the relationship between Kpov and other K2P channel proteins we generated the phylogenetic tree in *SI Appendix*, Fig. S2 *A* and *B*. The latter contains the 4 most similar channel proteins listed in *SI Appendix*, Table S1. In the absence of a full sequence of *P. orientalis* we further blasted the entire Kpov sequence or its pore-domain sequences separately against the NCBI data bank for *Pyramimonadophyceae*, *Pryamimonadales,* and Chlorophyta. This search was motivated by the fact that the known host of PoV1, the unicellular alga *Pyramimonas orientalis*, belongs to the order of *Pryamimonadales* and in the group of Chlorophyta (54). In 10 different *Pyramimonas* species, (*SI Appendix*, Table S3), we did not find a significant hit. Within the Pryamimonadales we found 3 candidate proteins with a K2P-like architecture from unicellular marine algae [K^+^ channel from *Ostreococcus tauri* (Kot), *Cymbomonas tetramitiformis* (Kct), and *Pycnococcus provasolii* (Kpp)] (*SI Appendix*, Table S1). Worth pointing out is that *C. tetramitiformis*, in which Kct was found, belongs with *P. orientalis* to the *Pyramimonadophyceae*; both species are hence closely related. Kpp from *P. provasolii* and Kot from *O. tauri* on the other hand are members of different classes (*O. tauri* belongs to the class *Mamoellophyceae* (55) and *P. provasolii* [recently reclassified as *Pseudoscourfieldia marina* in the class *Pseudoscourfieldiophyceae* (56)]; they are hence more distantly related. For comparison we also added the sequence of the two-pore-domain channel AtTPK1 from the plant *Arabidopsis thaliana* and the human K2P channel KCNK (TWIK1). The latter is considered the prototypical member of the class of K2P channels (5). (*SI Appendix*, Table S1).

In a multisequence alignment (*SI Appendix*, Fig. S2*A*) only the two filter sequences show an appreciable degree of conservation. A phylogenetic tree constructed from this alignment (*SI Appendix*, Fig. S2*B*) does not show apparent relationship between the viral channel (in red) and any of the other K2P channel proteins from algae (in green) or nonplant eukaryotes (in black). Taken together, the data suggest that Kpov is a unique protein with the structural hallmarks of a two-pore-domain K^+^ channel but with no obvious close relationship to any known K^+^ channel from other organisms. This also includes the K2P type channel Kct from *C. tetramitiformis*, the closest relative to the host of PoV1. The data imply that none of the pore units of Kpov is the result of recent molecular piracy by PoV1 from the host. The same conclusion was reached from a comparison of other viral K^+^ channels with K^+^ channels from their hosts (33, 34).

### Relationship Between Kpov and K^+^ Like Proteins from Related Viruses.

Gene sequences, which code for K^+^ channel-like proteins, have already been detected in many different types of viruses including some phages, but mostly in large dsDNA viruses (32, 57). However, while there are no reports on functional studies of putative K^+^ channels from phages, many studies have confirmed that giant DNA viruses in the phylum of Nucleocytoviricota contain genes that code for functional K^+^ channels (Fig. 1*C*) (25, 27, 32, 58–60). A common feature of viruses with genes for functional K^+^ channels is that they infect algae in freshwater and seawater habitats (26, 27, 59, 60).

Within the Nucleocytoviricota two orders, the *Algavirales* and the *Imitervirales,* contain viruses with K^+^ channel encoding genes. The *Phycodnaviridae* in the *Algavirales* are the largest source of viral K^+^ channels (Fig. 1*C*). While all the originally isolated Mimiviruses which belong to the *Imitervirales* infected amoeba, it was later found that newly found Mimivirus-related species also used seawater algae as their host. In a recent phylogenetic analysis, the additional families of *Allomimiviridae*, *Mesomimiviridae*, and *Schizomimiviridae* in the order of *Imitervirales* reveal the increasing diversity of Mimivirus relatives and account for their broad host range (Fig. 1*C*) (9).

While early reports on K^+^ channel like proteins were restricted to members of the *Algavirales*, data from recent genome analyses indicate that also algae infecting members of the *Imitervirales* contain genes encoding K^+^ channels (Fig. 1*C*) (27). In this context it is interesting to note that PoV1, which codes for the protein of interest here, is included together with the Tetraselmis-Virus 1 (TV1) in the family of *Allomimiviridae* (Fig. 1*C*) (9, 61). This family is only distantly related to the *Phycodnaviridae* (9). Genes which code for K^+^ channel-like sequences were already identified in several members of *Mimiviridae*-related viruses including a gene in TV1. Functional testing revealed that the latter channel protein with a two transmembrane domain architecture, named Ktv1, generated a K^+^-selective ion channel (27).

Because of this relationship between PoV1 and TV1 (Fig. 1*C*), we examined the similarity between Kpov from PoV1 and Ktv1 from TV1. This comparison was further extended to additional K^+^ channel-like proteins from other Mimivirus-like virus species (FloV-SA1, CsV-KB, Rhiv-SA1, and AAV1, see *SI Appendix*, Table S1, which infect marine algae and are classified within different families in the *Imitervirales* (Fig. 1*C*). The respective proteins have the structural hallmarks of K^+^ channels but turned out to be inactive as ion channels in electrophysiological assays (27). The selection was complemented by the sequence of the putative viral channels Kotu_6_1 and Kotu_6_2, which were recently discovered by metagenomic sea water analysis in unknown members of the NCLDVs (62). It has been mentioned before that the filter sequence of the respective Kotu_6_1 protein (*SI Appendix*, Table S1) shares a high degree of identity with the noncanonical filter sequence of the first pore unit in Kpov.

The functional Ktv1 channel protein and the other K^+^ channel-like proteins from NCLDVs were aligned with the two-pore-domain units P1 and P2 of the Kpov channel. The latter were composed of the first 81 amino acids for P1 from the N terminus to the short linker and the remaining 98 amino acids for P2. This separate comparison of P1 and P2 should answer the question of whether one or both pore units of the viral K2P channel share a relationship to K^+^ channel-like proteins in the related viruses.

*SI Appendix*, Fig. S3 *A* and *B* show the sequence alignment of the respective proteins and their corresponding phylogenetic relationships. The data indicate a high degree of identity between the two pore-forming units of Kpov; they are clearly distinct from the other proteins and form a separate branch. Their closest relatives are the recently discovered Kotu_6_1, Kotu_6_2 as well as the functional Ktv1 channel. These phylogenetic relationships are further underscored by a pairwise sequence alignment between the P1 and P2 domains of Kpov (*SI Appendix*, Fig. S4). It appears that 41% of their amino acids share the maximum BLOSUM62 score, suggesting a common ancestor for the two domains (63). Pairwise comparisons also reveal a significant amino acid identity of >30% between P1 and P2 on the one hand, and Kotu_6_1 and Ktv1 on the other. It is particularly striking that the unusual filter sequence of Kpov’s P1 is almost identical to that of Kotu_6_1 (*SI Appendix*, Fig. S4*D*). Taken together, the data suggest that P1 and P2 of the tandem Kpov channel, as well as the pore domains of some viral K^+^ channels with a conventional tetrameric architecture, descend from a common ancestor within the NCLDVs.

**Fig. 3.**
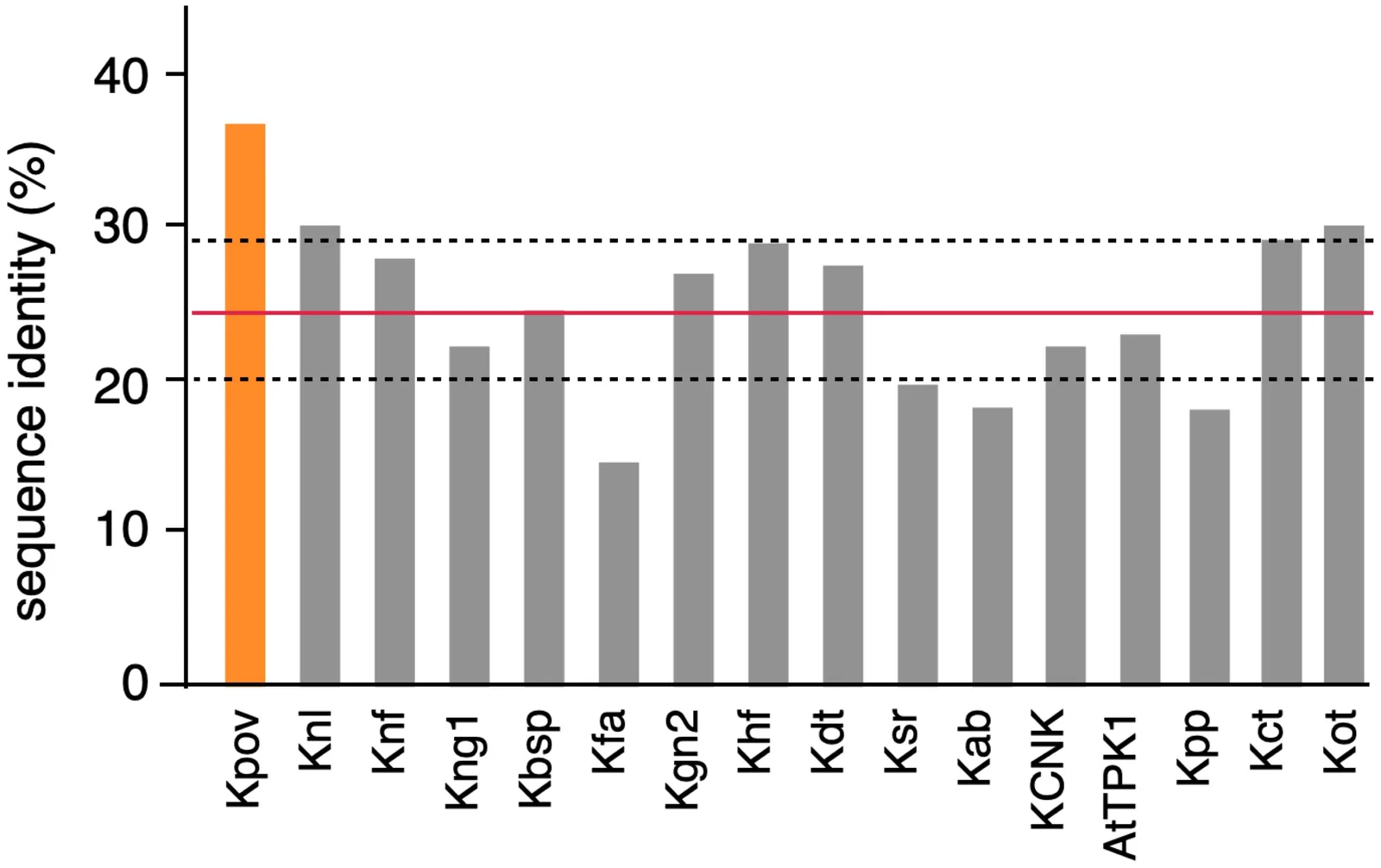
Similarity between P1 and P2 within selected K2P channels. The respective domains of P1 and P2 including the central GY/FG motif + 29 amino acids downstream and 51 amino acids upstream were pairwise aligned in CLUSTALW to obtain a value for sequence identity as percentage of identical residues relative to the total length of the alignment. The mean sequence identity values ± SD for mutual comparison between P1 and P2 domains of all nonviral K2P channels are indicated by red and dotted lines, respectively. The sources of the channel sequences are listed in *SI Appendix*, Table S1.

After realizing the high degree of similarity between P1 and P2 in Kpov, we asked the question on whether this is a unique feature of the viral channel or a general feature among K2P channels. We selected the four most similar sequences which contain two K^+^ channel filter motifs from *SI Appendix*, Fig. S2 and Table S1. We further selected K2P-type sequences from the protozooa *Naegkeria*, an evolutionary ancient organism believed to be ancestral to all eukaryotes (8, 64). Finally, we added the KCNK and AtTPK1 sequences as representatives of human and plant K2P channels, respectively. For a mutual comparison of the similarity between the respective P1 and P2 domains of each of the channels, we only considered the GY/FG motif + 29 amino acids downstream and 51 amino acids upstream of the common filter motif. Thus, the analysis is not biased by the different lengths of the domains. The respective sequences of the P1 and the P2 domains were subsequently aligned pairwise (*SI Appendix*, Fig. S5*A*) for each channel and analyzed with respect to their identity. The respective percentage of identical residues relative to the total length of the aligned sequences are shown in Fig. 3. The data indicate that the identity between P1 and P2 from Kpov is substantially higher than that of the other channels. The mean value ± SD of the alignment scores is 24 ± 4.8 for all the reference K2P channels. The number 37 from the Kpov channel is much higher than in the other channels.

This similarity between P1 and P2 of Kpov is further highlighted in a phylogenetic tree created from an alignment of all P1 and P2 domains (*SI Appendix*, Fig. S5 *A* and *B*) used in the analysis of Fig. 3. The tree indicates that the pore domains from Kpov are located on one common branch. The P1 and P2 domains of all other channels, apart from Kot, are more similar to domains in other channels than to their complementary tandem domain in the same channel.

The two current hypotheses regarding the origin of K2P channels suggest that they may have evolved either through gene duplication in early eukaryotes or through the fusion of two different genes in prokaryotes (23). The present data suggest a third possibility, namely that K2P-type channels may have evolved in viruses and been transferred to eukaryotic cells via viral gene transfer. The high degree of amino acid similarity between the two pore domains in the Kpov channel (Fig. 3), the lack of similarity to K^+^ channels from relatives of the viral host (*SI Appendix*, Fig. S3 *A* and *B*), and the rapid gating at hyperpolarized voltages typical for some viral channels (Fig. 2) indeed suggest a viral origin for both Kpov pore domains. This hypothesis predicts that the pores of Kpov should have a common ancestor within the family of viral K^+^ channels. If this is the case, they should also have a lower similarity to prokaryotic channels. To test this hypothesis, we constructed a phylogenetic tree from the two pore domains of Kpov, and a large number of K^+^ channel pores from DNA viruses; the latter contain distantly related Phycodnaviruses and Mimiviruses from freshwater and seawater habitats. For several of these viral proteins it was already confirmed that they are functional K^+^ channels [(26, 27); *SI Appendix*, Table S1].

**Fig. 4.**
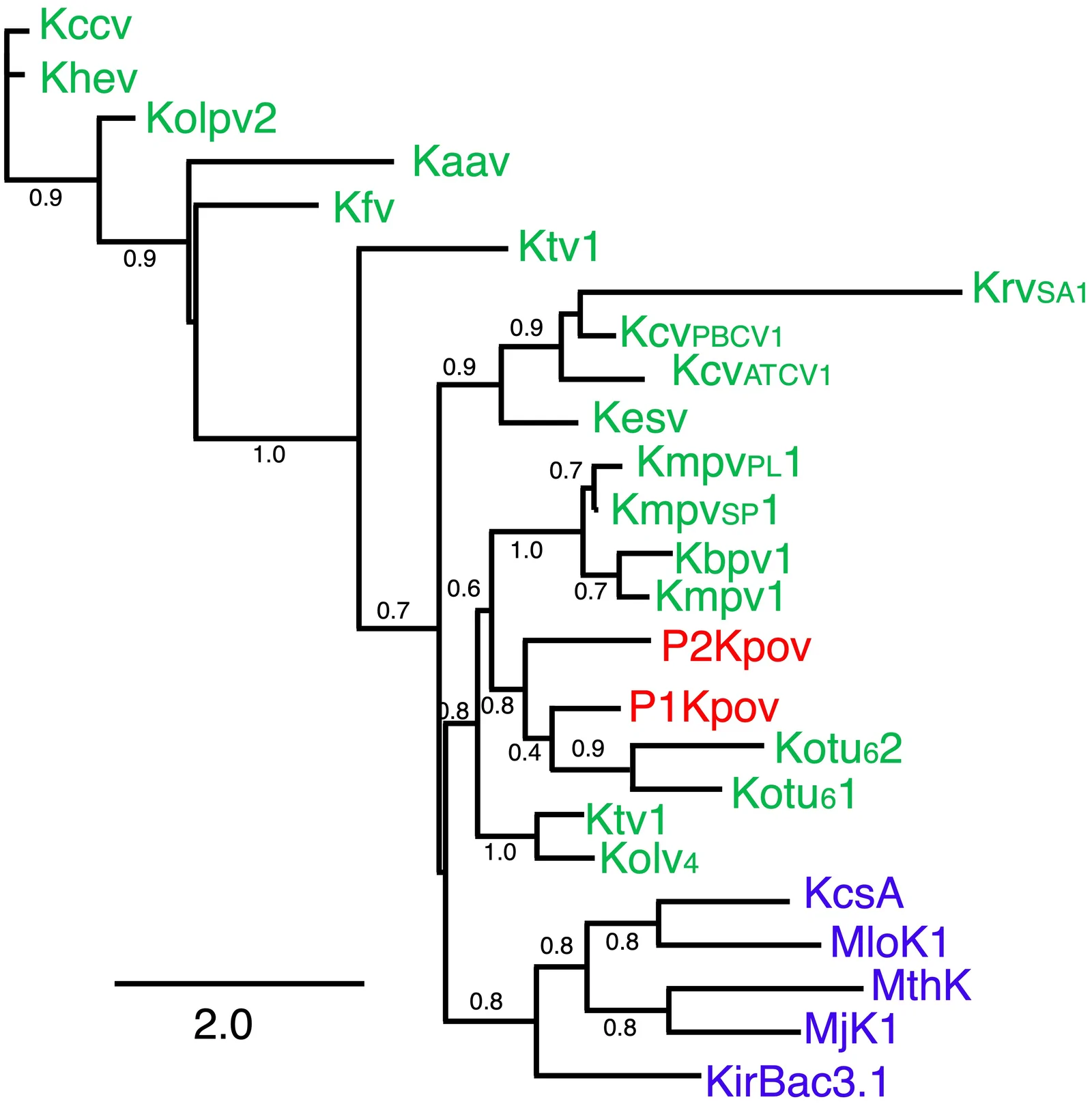
The two pore domains (P1 and P2) of Kpov are more similar to viral than prokaryotic K^+^ channels. Phylogenetic tree of representative bacterial K^+^ channels and viral K^+^ channels or K^+^ channel-like proteins. In blue: bacterial channels KcsA, MthK, MloK1, KirBac1.3, and Kjk1. In green: various functional and putative K^+^ channels from viruses. In red: P1 and P2 domains from viral K^+^ channel Kpov. The sources of all proteins are listed in *SI Appendix*, Table S1. The phylogenetic tree was constructed by the maximum likelihood method. Protein domain sequences were aligned with MUSCLE and the phylogenetic tree was created with PhyML and TreeDyn (www.phylogeny.fr). Branch support values are presented as numbers. Branch lengths reflect the number of inferred substitutions per site, as indicated by the bar.

The viral K^+^ channels were compared to the corresponding pore domains of representative prokaryotic K^+^ channels listed in *SI Appendix*, Table S1. The latter include the prototype K^+^ channel KcsA from *Streptomyces* lividans (gram-positive Eubacterium), MthK from *Methanobacterium thermoautotrophicum* (Archea), KirBac3.1 from *Burkholderia thailandensis* (gram-negative Eubacterium), MloK1 from *Mesorthizobium loti* (gram-negative Eubacterium) and MjK1 from *Methanocaldococcus jannaschi* (Archea). The alignment of the respective sequences (*SI Appendix*, Fig. S6) shows very little conservation. The prokaryotic and the viral K^+^ channels only have the short consensus sequence of the selectivity filter in common. A phylogenetic tree derived from this alignment (Fig. 4) reveals a very distinct separation between the prokaryotic channels and the viral channels. The two-pore-domain sequences from the Kpov channel are placed with strong statistical support within the viral channels. As expected, the closest relatives of the Kpov sequences are Kotu_6_1 and Kotu_6_2. Channels from viruses, which infect *Ostreococcus* algae in marine picoplankton, are the next closest relatives.

The phylogenetic analysis in Fig. 4 is in support of the view that the two pore domains from Kpov are most likely of viral origin. The data do not provide an answer to the question of whether the two pore domains are the result of a gene duplication or the consequence of a fusion of two independent genes for viral K^+^ channels. Notably, the amino acid identity between the two pore domains in Kpov is 41% (*SI Appendix*, Fig. S6*B*) not different from the degree of identity between K^+^ channels from different viruses (*SI Appendix*, Table S2). For example, the two functional viral channels Kmpv_1_ and Kotv_RT_ from different *Phycodnaviridae* show a similar low degree of identity (*SI Appendix*, Table S2). Hence, the sequence differences between the two pore domains in Kpov could well originate from the fusion of channel genes from different viruses.

In this context it is interesting to note that the GFG and the GLG motifs, which are frequently found in the filter of the second pore domain in animal K2P channels, are also present in different Kcv channels (25, 58, 65). Also, the length and amino acid sequence of the turret domain, the extracellular loop which connects the first TM domain and the pore helix, is very different between individual viral K^+^ channels (26). Hence, K2P channels from animals with a type of mixed filter sequences and an outer cap domain, could have also originated from the fusion of two different viral K^+^ channels. In fact, the respective long turret domain of the Kolpv_2_ channel (26), contains many cationic and anionic amino acids like the cap domain of animal K2P channels (66).

## Conclusion

Here, we show that the genome of the large dsDNA *P. orientalis* virus (PoV1), which infects a small unicellular marine green alga, contains a gene encoding the K2P-type channel Kpov. Like Ktv1 from the closely related Tetraselmis virus 1(Fig. 1*C*) (61), Kpov also exhibits robust ion channel activity, suggesting that genes encoding functional ion channels, albeit differing in structure, may be a shared feature within the family of Mimiviruses. Kpov exhibits a high unitary conductance of ~86 pS and a moderate, approximately 10-fold preference for K^+^ over Na^+^. Kpov is therefore classified as a K^+^-selective channel, although its preference for K^+^ over Na^+^ is significantly lower than that of eukaryotic K2P channels (44–47). As we currently do not know where and at what stage in the virus’s replication cycle the Kpov protein is expressed, it is impossible to speculate on the significance of such a reduced K^+^-to-Na^+^ selectivity in the K2P channel compared with canonical 2-TMD-type viral K^+^ channels.

The tandem channel is made from only 178 amino acids per monomer and is much smaller than all other known K2P channels; like the small 2-TMD type viral Kcv channel, Kpov also lacks any significant cytosolic termini. In addition to its miniature size, Kpov also shares with Kcv-type channels a peculiar fast and voltage-dependent gating at negative voltages (40). These common structural and functional features are compatible with a phylogenetic analysis showing that Kpov is unlikely the result of a recent molecular piracy from its host; its two pore domains seem to be most closely related to viral K^+^ channels.

Notably, such a phylogenetic reconstruction of K2P channels in which the two pore domains are of viral origin must be interpreted with caution. Because of the channel’s small size, alignments are only based on relatively short sequences. Also, the analysis considers only a limited sampling of eukaryotic channel genes; most importantly K^+^ channel genes from the host genome of PoV1 are not considered since the genome is not yet available. Hence, for the moment we can only conclude that the available data are consistent with a viral origin of Kpov.

This new aspect of the evolution of K^+^ channels, combined with the growing importance of large DNA viruses—in which we have detected the Kpov channel—for evolution (31, 35) suggests that the evolutionary history of K2P channels and K^+^ channels in general needs to be reassessed. A detailed analysis of the well-known mosaic genomes nature of these viruses (67), which harbor genes from various cellular sources, will reveal their role as an original gene source and/or as a vector for gene transfer between cellular organisms.

## Materials and Methods

### Sequence Analysis.

Primary sequences of putative K^+^ channels were compared with sequences in data-banks at a reference date of June 2026 using the BLASTP algorithm (https://blast.ncbi.nlm.nih.gov/Blast.cgi?PROGRAM=blastp&PAGE_TYPE=BlastSearch&LINK_LOC=blasthome). E values and amino acid identity (in %) of Blast searches with Kpov sequence as query are listed in *SI Appendix*, Table S1. Sequence alignment was performed with Muscle 3.7: (http://phylogeny.lirmm.fr/phylo_cgi/one_task.cgi?task_type=muscle) and phylogenetic trees calculated by PhyML3.0 algorithm (http://phylogeny.lirmm.fr/phylo_cgi/one_task.cgi?task_type=phyml) and visualized by treedyn platform (http://phylogeny.lirmm.fr/phylo_cgi/one_task.cgi?task_type=treedyn). The isolated pore domains P1 and P2 of different K2P channels were also aligned pairwise with ClustalW algorithm (https://www.genome.jp/tools-bin/clustalw) to obtain the percentage of identical residues relative to the total length of the alignment.

The structure of the dimeric pore was predicted by AlphFold2 (68) as implemented in ColabFold (https://www.nature.com/articles/s41592-022-01488-1).

### Protein Synthesis and Channel Recordings.

The K^+^ channels Kpov and Kcv_NTS S42T_ were synthesized cell-free with the MembraneMax^TM^ *HN* Protein Expression Kit (Invitrogen, Carlsbad, CA) in the presence of nanolipoproteins (NLPs) and purified as reported previously (39). The purified protein was functionally reconstituted in a vertical planar lipid bilayer as described previously (39). Planar lipid bilayer experiments were performed at room temperature (20 to 25 °C) with a vertical bilayer setup (IonoVation, Osnabrück, Germany). The recording chambers were prepared as described previously (39), and 1,2-diphytanoyl-*sn*-glycero-3-phosphocholine (DPhPC, Avanti Polar Lipids, Alabaster, AL) bilayers were formed using the pseudopainting/air bubble technique (42). For reconstitution of the channel protein, one of the elution fractions was diluted in 250 mM imidazole solution by a factor of 1,000 to 100,000, and a small amount (1 to 3 μL) of the diluted NLP/channel-conjugates were added directly below the bilayer in the *trans* compartment with a bent Hamilton syringe. After verifying the incorporation of a single channel in the lipid bilayer by short voltage pulses, constant voltages between +160 mV and −160 mV in steps of 20 mV were applied for 1 to 5 min. Both compartments of the bilayer chamber contained 100 mM KCl or NaCl solutions buffered with 10 mM HEPES; pH was adjusted to 7.0 with KOH or NaOH. The solutions were connected with Ag/AgCl electrodes to the head-stage of a patch-clamp amplifier (L/M-EPC-7, List-Medical, Darmstadt, Germany). Membrane potentials were applied to the *cis* compartment; the *trans* compartment was grounded. In the graphs, positive currents correspond to outward currents in the in vivo situation. Currents were filtered with a 1-kHz 4-pole Bessel filter and digitized with a sampling frequency of 5 kHz (LIH 1600, HEKA Elektronik, Lambrecht, Germany).

The reversal voltages (E_rev_) of Kpov and the reference Kcv channel Kcv_NTS S42T_ (40) were obtained from ramp protocols. Bilayers with active channels were first stepped for 5 to 10 s to +160 mV and subsequently subjected to a fast ramp (160 mV/s) to −160 mV. The open channel I/V relationships were obtained as follows: First, all current traces that showed no current fluctuations during the voltage ramp were identified and averaged. The control trace obtained in this way was then subtracted from all current traces that exhibited ion-channel-mediated current fluctuations during the voltage ramp. In this way, the capacitive currents were eliminated from the current traces. Finally, to obtain the I/V relationships, all corrected current traces were summed and plotted against the ramp voltage. The voltage range (ΔV ≈ 5 mV) in which the current inversion occurred was identified visually and subsequently fitted with a linear function providing the reversal voltage. Experiments were started with 100 mM KCl solution in the *cis* and 100 mM NaCl in the *trans* compartment, or vice versa. The NaCl solution was then gradually replaced with KCl solution to establish Na^+^ concentrations between 1 and 50 mM, keeping the total concentration of Na^+^ and K^+^ constant at 100 mM.

The E_rev_ values measured under these conditions were used to estimate the selectivity of channels for K^+^ over Na^+^ (P_K+_/P_Na+_) from the in the GHK flux equation:

$$E_{\mathrm{rev}}\;=\;\frac{\mathrm{RT}}{F}\;\cdot \;ln\left(\frac{{\left[{\mathrm{Na}}^{+}\right]}_{\mathrm{trans}}\;+\;\frac{P_{K+}}{P_{Na+}}\;\cdot \;{\left[K^{+}\right]}_{\mathrm{trans}}}{{\left[{\mathrm{Na}}^{+}\right]}_{\mathrm{cis}}\;+\;\frac{P_{K+}}{P_{Na+}}\;\cdot \;{\left[K^{+}\right]}_{\mathrm{cis}}}\right),$$

where R, T, and F are the gas constant, the absolute temperature at 25 °C, and the Faraday constant, respectively.

## Supplementary Material

Appendix 01 (PDF)

## Data Availability

Previously published data were used for this work (Protein data bank identity code QOI90491.1) (69).
