# Supplementary material for: A functional two-pore-domain K+ channel from a large DNA virus
Source: Proc Natl Acad Sci U S A. 2026 Oct 23;123(43):e2626158123. doi: 10.1073/pnas.2626158123 (PMC13715775; doi:10.1073/pnas.2626158123)
Supplement: Supplementary file 1 [file pnas.2626158123.sapp.pdf]

Supplementary Information

A functional two pore-domain K^+ channel from a large DNA Virus

Oliver Rauh[1*], Sebastian Mach[2*], Oliver Clarke[3,4], Jonas Toews[1], James L. Van Etten[5], Mike Althaus[1], Anna Moroni[6], Gerhard Thiel[2,6]

[1]Institute for Functional Gene Analytics, Department of Natural Sciences, Bonn-Rhein-Sieg University of Applied Sciences, Von-Liebig-Strasse 20, 53359 Rheinbach, Germany
[2]Membrane Biophysics, Technische Universität Darmstadt, 64287 Darmstadt, Germany
[3]Department of Anesthesiology, Columbia University Irving Medical Center, New York, NY, United States
[4]Irving Institute for Clinical and Translational Research, Columbia University, New York, NY, United States
[5]Department of Plant Pathology and Nebraska Center for Virology, University of Nebraska Lincoln, Lincoln, NE 68583-0900, USA; jvanetten1@unl.edu
[6]Department of Biosciences, University of Milan, via Celoria 26, 20133 Milan, Italy
*equal contribution

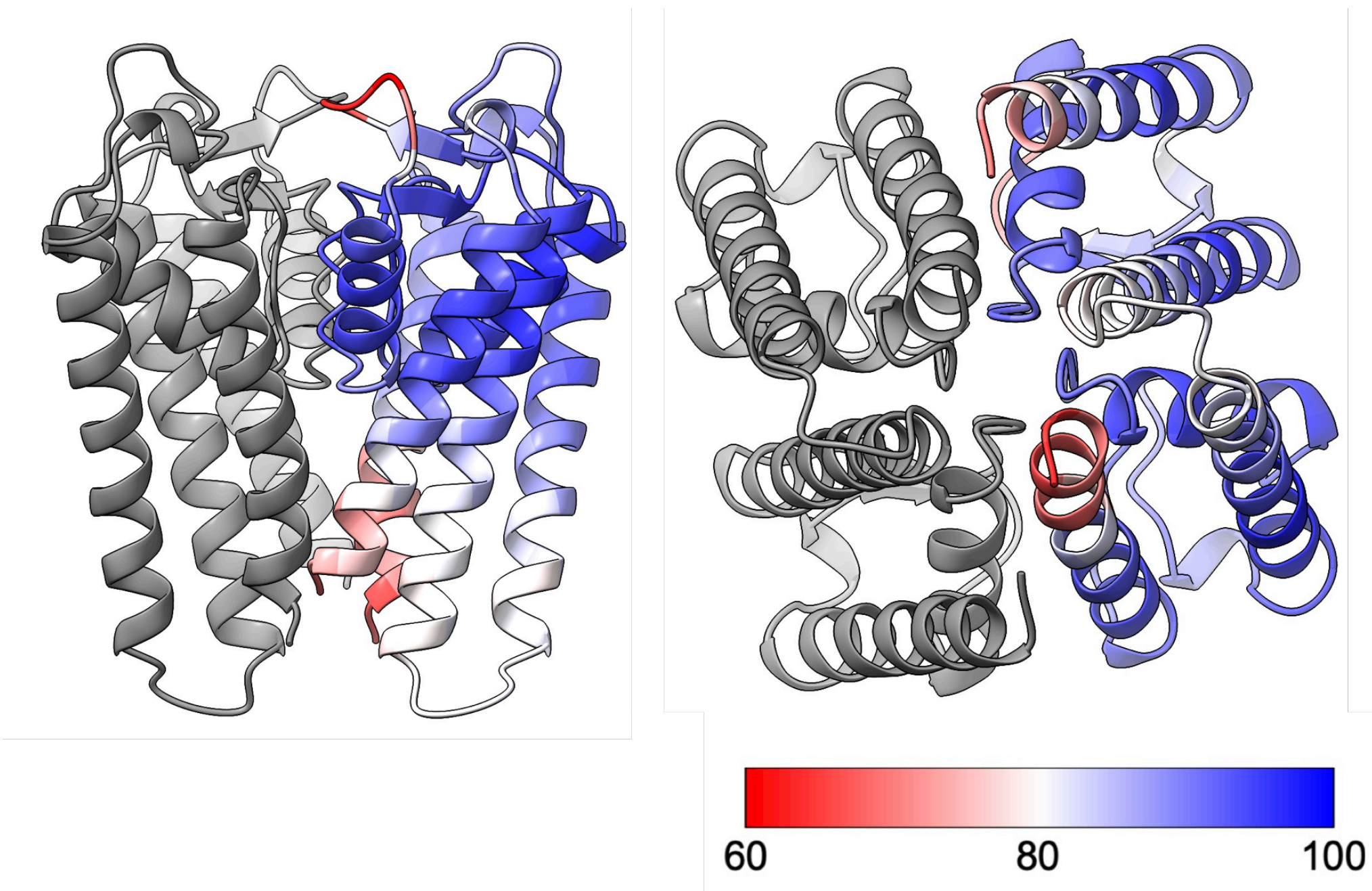

Supplementary Figure S1: Side view **(left)** and top view **(right)** of Kpov channel structure as predicted by AlphaFold2. The colors report for one subunit the per-residual confidence sore (pLDDT) between 60 and 100.

```
Kpov            MLl-------------------QILInfVViIFfatMI------FLNtdkTdwyaknykD
Khf             MVyvfqkssyggakmttpweqkKIVMglILgVFlyaLIgalafqYINplyTsqgldetyD

Kpov            lSFmDSlYlcttSfSSVGYGDIsPatkkSKIYv---IIIqtFILleiLsLAqYLsggFNI
Khf             tTFnDAfYfavvTlSTVGYGDVvPyddgTKLFtagyVLVslFLVgyaLeVSdFL---FSI

Kpov            gI----------------------LfNILItYfVILVATLYFtFVtDktdwnfpssndsn
Khf             dVeeleegneeetkrfmsrarkqlViNVLIvFlLMLVSTIFFvYVeD------------y

Kpov            SFLNmfYFTnTTVTTcGYGEIvPiTNKsRIpvMi---IqslIIlRVLs------------
Khf             SFVDslYFTvTTLSTvGYGDLaPrTHEgRLfaIfwvtIgvfMLfRLIgslmdvilesrkl

Kpov            ------------------------------------------------------------
Khf             rwraqreerqhvirehlsarvgiiamrnvtdteaiqhytkrdlievltkmefimyrleqe

Kpov            ----------------------------------LFT--------
Khf             gkvdlsdfrrldtafkdltpesinaefaspeserVFSsiignvvp
```

Supplementary Figure S2: Comparison between Kpov and the most similar K2P channel Khf
Pairwise alignment of Kpov with the most similar protein found by a Blast search of the non-redundant protein data base. The latter protein, named Khf, for K$^+$ channel like protein from *Hondaea fermentalgiana,* is annotated as a two pore-domain potassium channel 1 in the data base (**GBG27747.1.**). The sequence exhibits the typical tandem organization of the two K$^+$ channel filter motives with GYG motifs (in bold) in both proteins. In the alignment (performed with MUSCLE) similar residues are color-coded with an average BLOSUM62 scores of *Max: 3.0*, *Mid: 1.5*, *Low: 0.5*

A

```
Kpp           ----kalddllehhgsastkqrALarqackageatcgkmhysfdywhsW---ncLf---s
AtTPK1        phpskipmfsdlnpnlrrvImflalyltigtlcfylvrdqisghk--tsgvvDALYFciv
Kot           -----------makasevayqqlwilflvyifvailglqafdsssgaEfSFvDAfYFmai
Kpov          -------------mllqilInfvViiffatmiflntdktdwyaknykDlSFmDsLYlcTT
Kct           -----flklvrripplttaLlkiVlfyllgmvfyyete---------gWSvfEcaYFtTv
Khf           ---qkkivmglilgvflyaLigALafqyinplytsqgldety-----DttFnDAfYFAvv
Kdt           -----kmllgvvalltaylVggAIifryinpqfrtqgnnaef-----EttFlDAMYFvvv
KCNK          -rrfleeheclseqqleqfLgrvLeasnygvsvlsqasgnw------NWdFtsALfFAST
Ksr           ---lgaidnaiadgnfttnqtqvLmnlldtldelrstavnigkt---DWSFvgAtYFATT
Kab           ---------------idrlIqtAMeafsegiqpqdvsfdavqtlrkpkWtFqtALYFSvT

Kpp           TITTIGYGnIaPaTpeGraFlLiYsLIGfvLngVtLttmStlinrfst------------
AtTPK1        TMTTVGYGDlVPnSsasrLlacafvfsGmvLVghlLSraADYLvekqeAllvrAfhlrQs
Kot           TvTTVGYGDItPtTDkGKVFmIfviIsGIsLatVviSkItDliisakeAselaAqErlEq
Kpov          sfssVGYGDIsPaTkksKI----YvIIiqtfIlLeilslAqYL-----------------
Kct           TIsTVGYGDIsPtrDisKLFtcaYmcIvL-------------------------------
Khf           TLsTVGYGDVVPydDgtKLFtagYvLVsLfLVgyaL-eVSDFLfsidv-------EelEe
Kdt           TLTTVGYGDIVPsnDetKIFvslYiIVsLvLVgVaL-eVtDFLfqidv-------EeiEe
KCNK          vLsTtGYGhtVPlSDgGKaFCIiYsVIGIpftlLfLtaVvqritvhvtrrpvlyfhirwg
Ksr           avTTIGYGwIaPkTvgGrVFCIlYsVIGIpLVfymfAylgrkmmdiig---frisslrEg
Kab           iLTsVGYGnlVPiSrlGrIFCIvYgtfGIpLtlItiAdmAkFLsdlia--raeAkykqik

Kpp           --ylntlid----------------------------lplvseq-YenvfLisIatmah
AtTPK1        fgptDilkelh--------------------------tnklrykcYatcLVlvVLfiVg
Kot           smekDlmmlrqklgnilsaedlsrfsedaksghdesaaphpvvrvVYhpvsViiIVLlIg
Kpov          --------------------------------------sggfnigIlfnILItyfVIlVa
Kct           ------------------------------------------FigIVIgmtVLdln
Khf           gneeEtkrf----------------------------msrarkqLvinvLIvfLLMlVs
Kdt           ggevEldkf----------------------------ltrgrkrLmwnLLwvfLfMfss
KCNK          fskq---------------------------------vvaivhaVllgfVtvscfffIp
Ksr           seykrkqlq----------------------------sdsvvlpMFvaLfIaaLLIsVf
Kab           tddeDvlae----------------------------pgpwsktfvlfLLLsyMaIsav

Kpp           vIFyslImmDTEgWDmptS---------vYFSwVTfTTIGfGDYyP----TNEga-kavg
AtTPK1        tIFlVmVEk----mpvisa---------FYcvcsTVTTlGYGDksf--n-sEagR-Lfav
Kot           AatFcaVEpE---iSYlDg---------vwwavVTsTTVGYGDilP--t-TDKaK-Ifas
Kpov          tLYFtFVtdkTD-WNFpsSndsnsflnmFYFTntTVTTcGYGeivP--i-TNKsR-Ipvm
Kct           -------------W--vDS---------tYFifVTasTVGYGDviPvgk-sDlsK-Vlvs
Khf           tIFFVYVEd----ySFvDS---------lYFTvtTLsTVGYGDlaP--r-ThEgR-Lfai
Kdt           vIFFVFqDeEs--ftavDa---------iYFTvaTMTTVGYGDFtP--r-sDEgR-Ifsi
TWIK          AavFsvLEdD---WNFlES---------FYFcfIsLsTIGlGDYvPgegynqKfReLyki
Ksr           AIaFtYtEt----WtYfES---------FYFvfITMTTIGfGDFvPtyr-dhpvp-Lilq
Kab           AcaF-FkEt----WNFlDS---------FYFclITMTTIGYGDldP--alavEtp-dhyg

Kpp           gya---rYIMIntimtMtglsilasllgala-
AtTPK1        fWi---ltstIcLaqfflyVaelntenKq---
Kot           fya---lFVVgvMgwaVsqI------------
Kpov          iiq---slIILrvlslft--------------
Kct           cfi---liLVVtLadaIhhVhtinvrrRir--
Khf           fWvTiGvFmLfrLigsLmdVil----------
Kdt           fWvSaGclVLfrcvgsLmdVmlearkrKwtks
KCNK          git---cYLLLgLiamLvvletfcel------
Ksr           vfg---iFLaLsvysyLinVaivlvt------
Kab           gWi---aFIfVgLilstltVdmcgss------
```

B

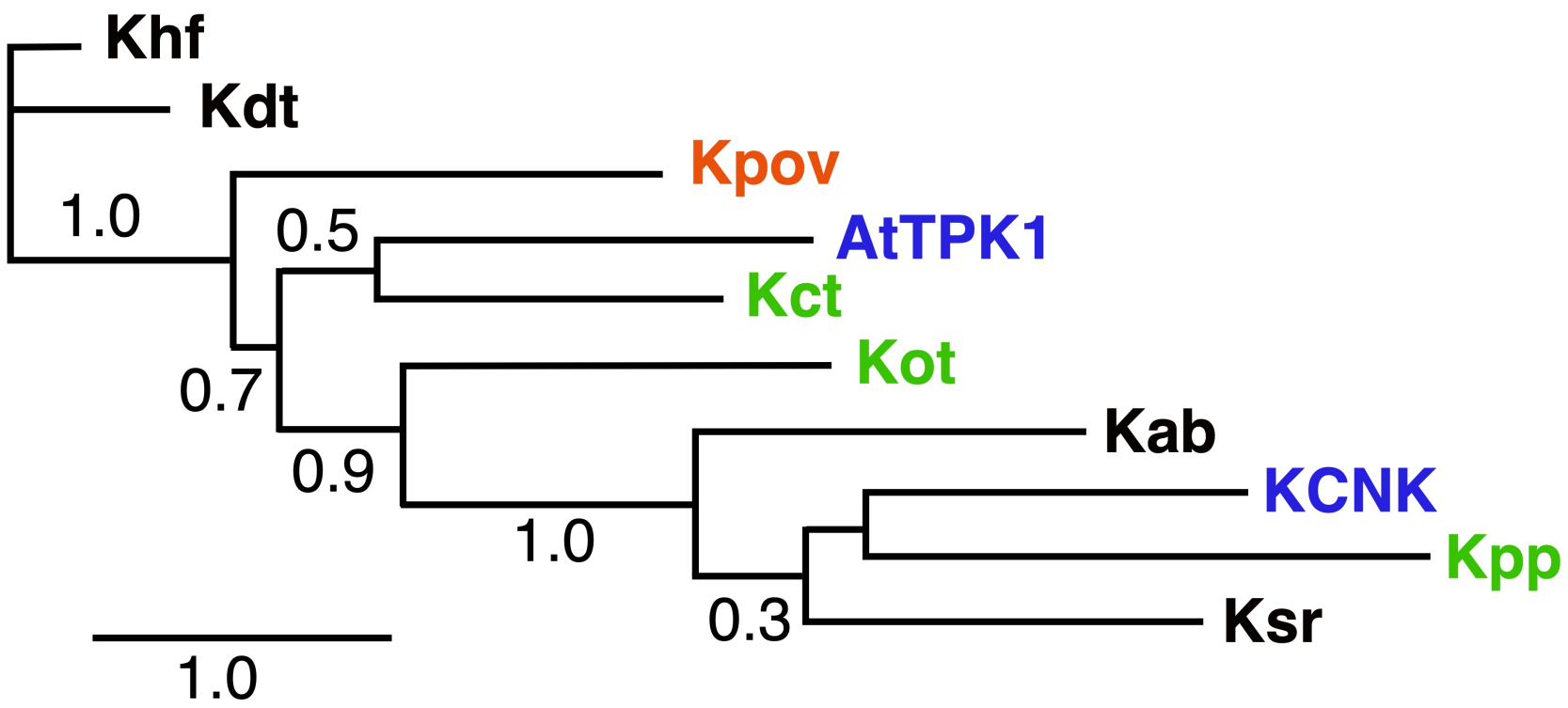

Supplementary Figure S3.

A: Multiple sequence alignment of Kpov with K2P channel sequences from other organisms (Supplementary Table 1). Alignment of viral protein with K2P channels from different organisms (Supplementary Table 1) was performed with MUSCLE after deleting in non-viral proteins parts of the long N- and/or C- termini to favor alignment of the second GY/FG filter motifs. The eight amino acids of the filter motifs are in bold. In the alignment similar residues color-coded with average BLOSUM62 scores of *Max: 3.0*, *Mid: 1.5*, *Low: 0.5*

B: Phylogenetic tree for Kpov and other K2P channels from alignment in A. Each defined node in the tree is labelled with its corresponding bootstrap confidence level. The tree was constructed from the alignment in Figure 1S supplement including the 4 most similar proteins to Kpov (Kdt, Khf, Kab, Ksr from primitive eukaryotes, in black), three K2P-like channels (Kpp, Kot and Kct) identified in algae (Chlorophyta and Pyramimodales, in green) as well as a higher plant (AtAKT1 (KCO)) and human K2P channel (KCNK(TWIK)) (in blue). Phylogenetic tree was constructed by maximum likelihood method. Protein domain sequences were aligned with MUSCLE and the phylogenetic tree was created with PhyML and TreeDyn (www.phylogeny.fr). Branch support values are presented as numbers; branch lengths reflect the number of inferred substitutions per site, as indicated by the bar.

A

```
KrvSA1          --------minrikaIrF----IffnlicsfiFgVLYyLVqfie---enafLhpI-----
Ktv1            ---------------msF-vaKVaVhiilIItFfVInltInitn----------------
P2Kpov          --sLaqyLsggFnigIlF---nIlItYFvILvaTLyFtfVtdktDwN-------------
P1Kpov          ---------------mll---qIlInFvvIIFFAtMifLntdktDw--------------
Kaav            -------------mkLlFgYNRfhLliYqIIFFSILYmfLgssH-FsGINtLEDIlKnEI
KfvSA1          ---mlfNkKniFyknIys---KIiIlilsfIlFgILYyfLcddYeFgGINiLQEeIrnss
KccvKB1         MSdIfyNIKtnFqnnIsY-FNKIqIlFFlMfvFSLLYmLLddeH-FeGVNkfKEIVKEEV
Khev            MNtIanNIqknihsltkFriNKIkIllFsMLFFSLvYmLLddrH-FeGVNkfKELVKEEV
Ktou6-1         -------MKltiikkIik---slffvFllsLcFSIvlsLfdytH-wNGIde---------
Ktou6-2         -----mfVKsivldkIgv---sllLytvaItvtAfflsfLdftH-FNGfeieR-------

KrvSA1          ------------------------------------------------------------
Ktv1            ------------------------------------------------------------
P2Kpov          ------------------------------------------------------------
P1Kpov          ------------------------------------------------------------
Kaav            VskqVldpIiEekftnasDpskFiSKddIevd----------kkEtEeiqEkAKEIkKEV
KfvSA1          lKkfVdKiesE-------------------------------qlnKEVksEisekIsKEi
KccvKB1         IKDkakQeIkENYdNlKE----yYndNKIinktkfsennsnniiEEkViDEaAKktEqEV
Khev            IKDkVkKkIiENYsNlKDDletFYSKNK--------------vikEkVfDqaAKEtEKEV
Ktou6-1         ------------------------------------------------------------
Ktou6-2         ------------------------------------------------------------

KrvSA1          ---ELKlyisKnNkkrsllrcIYFglvTqTsIGYGgfvPygpiassLnaLQmvsifi-In
Ktv1            --------knafskelttlDvfYytvTTwTTtGYGDIyPvitvSKmIAvtKmllfLv---
P2Kpov          --------fpssNdsnsFlnmfYFtnTTvTTcGYGeIvPitnkSripvmIiqsliIlrV-
P1Kpov          --------yaKnykdlsFmDsLYlctTsfssvGYGDIsPatkkSKiyviIiqtfiL----
Kaav            KK-ELniltdKdsffdRFFlRfYFsfvTsTTIGYGDttPsSistrtLAmIQacsTfy---
KfvSA1          rhpsiEeavpKsNrlqKiFDRIYFsvvTgTTLGYGDIyPlSnklKsLiiIQlftTIt---
KccuKB1         KKEELsiekvepNlikKYFDRLYFaiiTgclLGYGDIfPvtytSKtLAgIQgllTVs---
Khev            KKEELQpdkiepsnidKYFnRLYFaivTgclLGYGDIyPvtlaSKlLtgIQgllTVs---
Ktou6-1         --------kedeNmitKilnRLYFvtTTmssvGYGDIsPkShitKiLvimmqmfvViGf-
Ktou6-2         --------veKhsllqRYFDRLYFcsTTfsTvGYGDItPkSniaKfLtiLlqfiStiGIi

KrvSA1          SlIIF-------
Ktv1            -ILmY-------
P2Kpov          -lsLFt------
P1Kpov          -leIl-------
Kaav            -ILma-------
```

```
KfvSA1          -ILfs-------
KccvKB1         -lIIY-------
Khev            -lIIY-------
Ktou6-1         -IaIYqskiigl
Ktou6-2         SIIVF-------
```

B

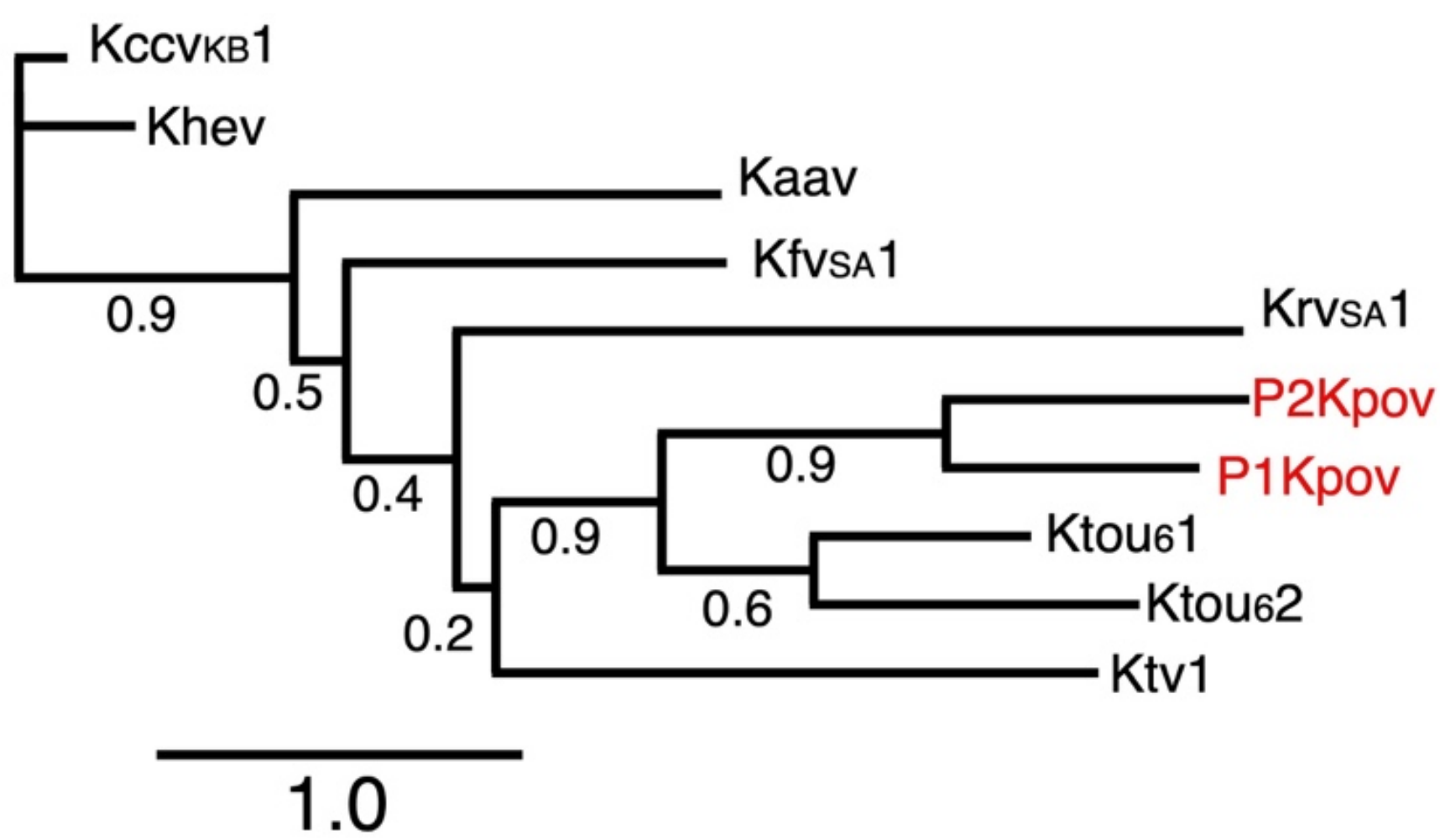

Supplementary figure S4:

A: Similarity between Kpov with K^+ like sequences from related *Mimiviridae* viruses. Multiple sequence alignment of the two pore domains (P1/P2) from Kpov with K^+ channel Ktv from TV1 and K^+ channel like sequences from other large DNA viruses classified in the order *Imiterales* (Supplementary Table 1). The eight amino acids of the filter motifs are in bold. In the alignment similar residues color-coded with average BLOSUM62 scores of *Max: 3.0*, *Mid: 1.5*, *Low: 0.5*

B: Relationship between the two pore-domains (P1, P2) from Kpov to K^+ channel or K^+ channel like proteins from viruses related to PoV from alignment in A. Phylogenetic tree of P1 and P2 from Kpov with functional K^+ channel Ktv1 from Tetraselmis virus 1 and putative K^+ channel like proteins from viruses in the order *Imitervirales* (Supplementary Table 1). Phylogenetic tree was constructed as in Fig. S2 Branch support values are presented as numbers; branch lengths reflect the number of inferred substitutions per site, as indicated by the bar.

A

P1Kpov llqILInFvVIIffatmiflnTDKTDW-YakNykdlSFMDslYlctTSfSSvGYGDIsPa
P2Kpov -fnILItYfVILvatlyftfvTDKTDWnFpsSndsnSFLNmfYftnTTvTTcGYGEIvPi

P1Kpov TkKSKIyVIIIQTfILLeILSLaq
P2Kpov TnKSRIpVMIIQSlIILrVLSLft **(41%)**

B

P2Kpov slaqYLSggfnIgIlfnILITYFVIlVatlyftfVTDKtdWnfpssNdsnSfLNMFYFTn
Ktv1 --msFVA---kVaVhiiLIITFFVInLtin----ITNKnaF-----SkelTtLDVFYYTv

P2Kpov TTvTTcGYGEIvPItnkSRIpvMiIqsLIIlrVLsLFt
Ktv1 TTwTTtGYGDIyPVitvSKM--IaVtkMLLflVIlMY- **(38%)**

C

P1Kpov ---LlQIlINfVVII-FFatmIfLNtdktDwYAknyKDLSfMDslYlctTSFSSvGYGDI
Ktv1 msfVaKVaVHiILIItFFvinLtINitnkNaFS---KELTtLDvfYytvTTWTTtGYGDI

P1Kpov sPatkkSKIyViIiQtfIlLeILslaq
Ktv1 yPvitvSKM-IaVtKmlLfLvILmy-- **(33%)**

D

P1Kpov ----LLQiLInfVviIFfaTMiFlnt----DkTdWyakNyKD-----lsfMDsLYlcTT
Kotu$_6$1 mkltIIKkIIksLffVFllSLcFsivlslfDyThWngiDeKEdenmitkiLNrLYfvTT

P1Kpov SfSSVGYGDISPaTkkSKIyVIIIQtFILLeiLSLaQ------
Kotu$_6$1 TmSSVGYGDISPkShiTKIlVIMMQmFVVIgfIAIyQskiigl **(36%)**

E

P1Kpov fnIlI------TyFvIlVaTLyFTfVt---DkTdWN-fpssnDsN---sfLNmfYFtnT
Kotu$_6$1 mkLtIikkiikSlFfVfLlSLcFSiVlslfDyThWNgidekeDeNmitkiLNrlYFvtT

P2Kpov TVTTcGYGEIvPiTNkSRIpVMIIQslIILrvLSLFt------
Kotu$_6$1 TMSSvGYGDIsPkSHiTKIlVIMMQmfVVIgfIAIYqskiigl **(35%)**

Supplementary Figure S5: Mutual similarity between the two pore-domains P1 and P2 of K2P Kpov channel and between these pores with channels Ktv1 and Kotu$_6$1. Pairwise sequence alignment of the pore domains P1 and P2 from Kpov **(A)** and pairwise alignment of Ktv1 with P1 **(B)** and P2 **(C)** as well as alignment of Kotu$_6$1 with P1 **(D)** and P2 **(E)**. The eight amino acids of the conserved K^+ channel filter motifs are in bold. For this mutual comparison of P1 and P2 domains we only considered the GYG motif plus 29 amino acids downstream and 51 amino acids upstream of the common filter motif to avoid a biased by the different lengths. In the alignment of similar residues is color-coded with average BLOSUM62 scores of *Max: 3.0*, *Mid: 1.5*, *Low: 0.5*. Numbers in brackets provide percentage of amino acids with max. BLOSUM62 score relative to the total number of amino acids in each alignment.

A

```
P2Kpp        -----lplvseqyenVflisiatmahvifYsLLImmdtE------------gWdm------
P1Kpp        -dllehhgsastkqralarqackageAtcgk----mhy------------sfdy------
P2Kpov       -------------fnIlityfvilvatlyFt----fVtdkt---------DWNFpSsnds
P1Kpov       --llqilinfvVii-ffatmiFlntdktdw-----yaknyk---------DlSF------
P2Kfa        nrkaglavqilMyl-apgfvmFiffpAflFs----yyE------------EWSy------
P2Kbsp       -rvkvmvkrkmIvrmIalwivFwfggAgvFc----afE------------EWTy------
P2Ksr        -qlqsdsvvlpMfvaLfiaallisvfAiaFt----ytE------------tWTy------
P2AtTPK1     -elhtnklrykcyatclvlvvlfivgtiflv----mVE------------kmpv------
P1Kab        ------aidrlIqtaMeafsegiqpqdvsFd-----aVQtlrkP-------kWTF------
P2KCNK       --kqvvaivhaVllgfvtvscFffipAavFs----vLEd-----------DWNF------
P1KCNK       eclseqqleqfLgr-VleasnY---gvsvls----nasgnw---------NWdF------
p1Ksr        -----adgnfttnqtqvlmnlldtldelrst----aVnigkt--------DWSF------
P1Kfa        -------esseaqhdVilkkltdycgksvYn----ytEteevPl------kWdF------
P2Khf        krfmsrarkqlVin-Vlivfllmlvstif Fv----yVE------------DySF------
P2Kdt        --fltrgrkrlMwn-Llwvfl Fmfssvif Fv----fqD------------EeSFtA----
P2Kab        --vlaepgpwsktfvLflllsYmaisAvaca----ffke-----------tWNF------
P1AtTPK1     --------npnLrrvImflalYltigtlcFyLVrdqIsghk---------tsgv------
P1Kbsp       saglsatlkalIis-sfimtiviivgAmvYt----wLE------------gWTF------
P1Kng1       aqtlnqmtsnftinnVttssnetditydlFn-----ID------------hWyy------
P1Knf        ipiwaieggneLkq-Mrennitltnennsit----qLE------------kWNy------
P1Kng2       -----laykenLlnstldeysFdnstgnanv----tIDpfnp--------kWTy------
P2Kng1       svivkhplsqiVyf-fflvatYsivgAliFm----afE------------DWvF------
P2Knf        ykflshlltqfsyy-tffwlaYvfvcAaiFm----aIE------------gWSy------
P2Kng2       fsffnrgatqiInm-IlllggYvvgaAalFs----yLE------------NWeF------
P2Kot        --aaphpvvrvVyhpVsviiivlligAatFc----aVEp-----------EiSy------
P1Kot        ---asevayqqLwilflvyifvailglqaFd----sssga----------EfSF------
P1Kdt        -------------gvValltaYlvggAiiFr----yInpqfrtQGnNaeFEtTF------
P1Khf        -------------glIlgvflYaligAlaFq----yInplytsQGlDetYDtTF------

P2Kpp        ---ptSvYFswVTfTTIGfGDyyyPtnegaKavggYaryImIntImtMtg-
P1Kpp        ---whSWnclfsTITTIGYGnIaPaTpeGraFlliYsLIGfvLngvtLt-
P2Kpov       nsflnmFYFtntTvTTcGYGeIvPiTnksrIpvmiiqsLiIlrVlsLft-
P1Kpov       ---mDSlYlCttsfssVGYGDIsPaTkksKIYviiiqtfiLleIlsLaq-
P2Kfa        ---dEavYyafVTLTTIGfGDyvagqdntKgsgfFFiMyksfLIawIsf-
P2Kbsp       ---lESlYFCyVTLTTIGfGDyvPtepgsieFwniYvfIGLtIfayILs-
P2Ksr        ---fESFYFvfITMTTIGfGDfvPtyrdhpVplil-qVfGIfLalsVysy
p2AtTPK1     ---isavYcvcsTvTTlGYGDksfnseaGrLFavFwiLtssicLaqffl-
P1Kab        ---qtalYFsvtiLTsVGYGnLvPisrlGrIFcivYgtfGIpLtlitIa-
P2KCNK       ---lESFYFCfIsLsTIGlGDyvPgegynqkFrelYkIgitcyLllgLi-
P1KCNK       ---tsalfFastvLsTtGYGhtvPlsdgGKaFciiYsVIGIpftllfLt-
P1Ksr        ---vgatYFattavTTIGYGwIaPkTvgGrVFcilYsVIGIpLVfyMfa-
P1Kfa        ---ynSFYFaytvvsTIGYGnLaPtnslGrIlmiFYgLIGIpMngiLLa-
P2Khf        ---vDSlYFtvtTLsTVGYGDLaPrTheGrLFaiFwvtIGVfMLfrLIg-
P2Kdt        ---vDaiYFtvaTMTTVGYGDftPrsdeGrIFsiFwvsaGclVLfrcvg-
P2Kab        ---lDSFYFClITMTTIGYGDLdP-alavetpdhYggwIafifVglILsa
P1AtTPK1     ---vDalYFCiVTMTTVGYGDLvPnssasrLlacaFvfsGmvLVghLLs-
P1Kbsp       ---dDSvnFCiVsfsTIGYGnLsPrsvaGrIFffFYgfLGVsaIgffIv-
P1Kng1       ---tnclYFstVTfTTVGYGDVvPqTvaGKLFvvlFgILGLatMgaMtg-
P1Knf        ---gnSvYyClVTfTTIGYGDfyatTtgGrVYigFFgLaGIaIMgsLLv-
P1Kng2       ---gnSvYFmiVTLsTIGYGDMsPtTigGKLwvvFFgfLGIsLMgmwIs-
P2Kng1       ---gDSlYFvfITLTTIGYGDLklknsgsKfFliFFiIcGIgLLgiLLa-
P2Knf        ---gDacwyCfVTLgTIGYGDyyPkTrgGKVFfmFFaIIGLgfeaiMIg-
P2Kng2       ---yDaFYysfVTLsTIGYGDfyPkTtnGKItfgFFvLIGLgLLgiLLg-
P2Kot        ---lDgvwwavVTsTTVGYGDIlPtTdkaKIFasFYaLfvVgVMgwavs-
P1Kot        ---vDaFYFmaITvTTVGYGDItPtTdkGKVFmiFviIsGIsLatvVIs-
P1Kdt        ---lDamYFvvVTLTTVGYGDIvPsndetKIFvslYiIVsLvLVgvaLe-
P1Khf        ---nDaFYFavVTLsTVGYGDVvPyddgtKLFtagYvLVsLfLVgyaLe-
```

B

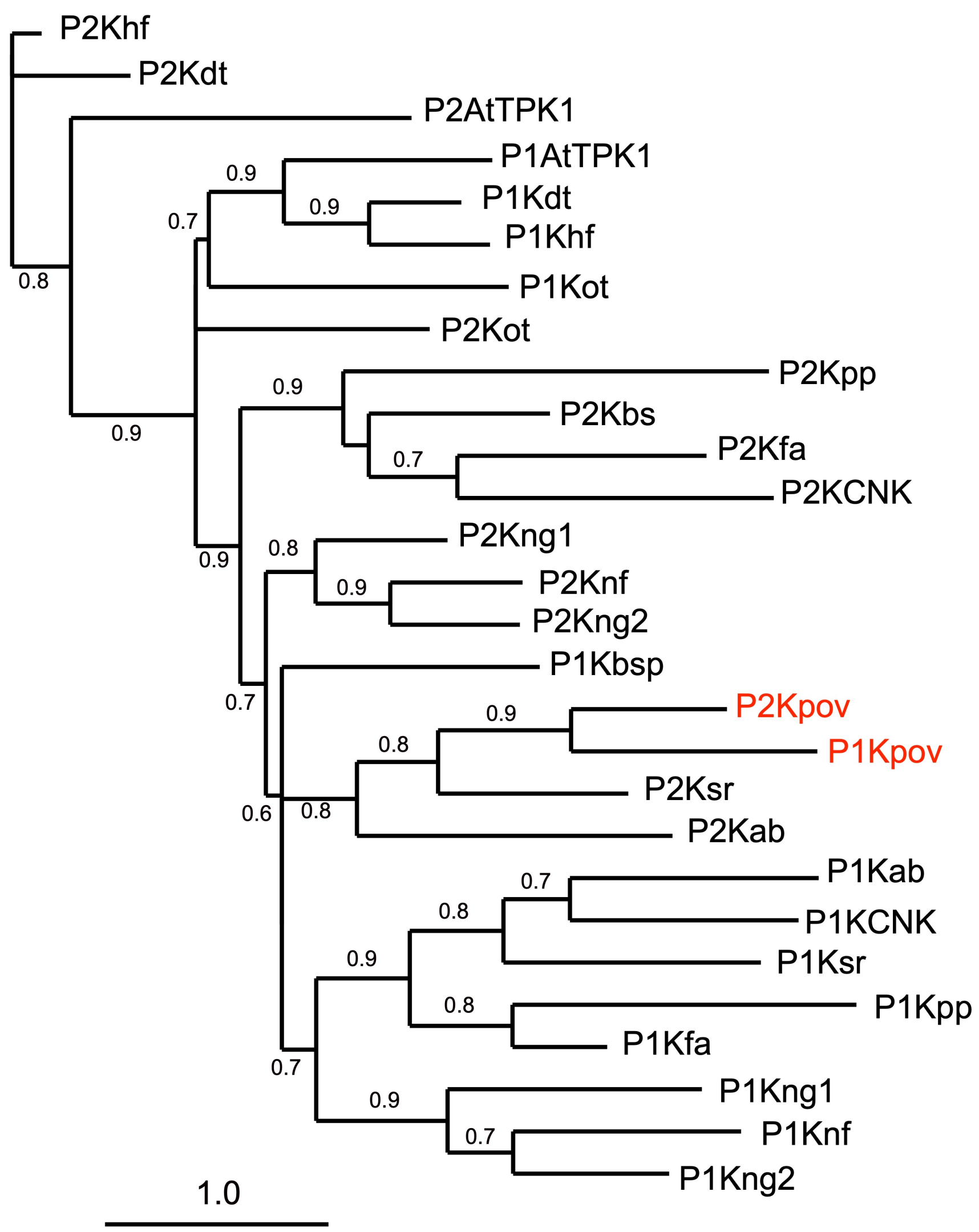

Supplementary Figure S6:

A: **Similarity between the two pore-domains P1/P2 in different K2P channels**. Multiple sequence alignment of the two pore domains (P1/P2) from different K2P channels. Information on the source of the sequences is found in Supplementary Table 1. Alignment is performed with Muscle of the pore domains with the two GY/FG motifs plus 29 amino acids upstream and 51 amino acids downstream of each filter motif. The eight amino acids of the filter motifs are in bold. In the alignment similar residues color-coded with average BLOSUM62 scores of *Max: 3.0*, *Mid: 1.5*, *Low: 0.5*

B: Phylogenetic relationships between the two pore-domains in different K2P channels from alignment in A Phylogenetic tree of P1 and P2 domains from different K2P channels. The tree was constructed by comparing in each case a sequence of 80 amino acids including the GY/FG motif plus 29 amino acids downstream and 51 amino acids upstream of the conserved filter motif. The source of channel sequences is listed in Supplementary Table 1. The pores P1 and P2 from Kpov are highlighted in red. Each node is labeled with its corresponding bootstrap confidence level. The tree was constructed as in Fig. S2. Branch support values ≥ 0.5 are presented as numbers; branch lengths reflect the number of inferred substitutions per site, as indicated by the bar.

KrvSA1 -----yylvqfieenaflhpielklyisknNK-kr----SlLrcIYFgLvTqtsIGYGgf

MthK	--lkvpatrILllvlaviiygtagfhfiegE---------SwtvsLYwtfvTiaTVGYGDy
Kjk1	-tyekielgIIviillililiesvilmtvegw---------dfftafYtaVvTiSTVGYGDy
KcsA	--atvlLviVLlagsYlavlaergapgaqli---------TypraLwwSVeTatTVGYGDL
MloK1	vttlfgVvlfavalaayvierdiqpEkfg----------SipqaMwwaVvTlSTtGYGDt
KirBac3.1	laalfvVnntLFallYqlgdapianQspp----------gfVgaffFSVeTlaTVGYGDm
**P2Kpov**	-----yfviLVatlyFtfvtdktdwnfpssNd-sn----SfLnmfYFtnTTvtTcGYGeI
**P1Kpov**	----nfVviIfFatmiflntdktdwyakny-K-Dl----SfMDsLYlctTsfSsVGYGDI
Kaav	eeiqekakeIkkevkkelniltdkdsffd----------rfflrfYFSfvTstTIGYGDt
Kfv	--vkseIsekIskeirhpsieeavpKsnrlq--------kifDrIYFSVvTgtTlGYGDI
Kolpv2	pynkekVkedVknivrneedkiykpnyfq----------ryLDsLYFSIiTscllGYGDI
KccvKB1	----------VideaakkteqevkkEelsiEKvEpnlIkkyfDrLYFaIiTgcllGYGDI
Khev	----------VFdqaaketekevkkEelqpDKiEpsnIdkyfnrLYFaIvTgcllGYGDI
Ktv1	---avhIilIItffvinltinitnknafskEl-------TtLDvfYytVTTwtTtGYGDI
$Kotu_{6}2$	-vtaffLsfLdFthfngfeiervekhsllq---------ryfDrLYFcsTTfSTVGYGDI
$Kotu_{6}1$	-slcfsIvlsLFdytHwngideke--denmit-------kiLnrLYFvtTTmSsVGYGDI
Kolv4	-----fVtiLIFamfYyflaltggdhfngiDs-Nv----SlMDafYFSsTiqSTVGfGDI
$Kotv_{RT}$	-----fIsiLLFtlmYftiskaggeQfnglDK-Ds----gfLDhLYFafTvqSTVGfGDI
Kcv_{ATCV}	lliihiIilIVFtaiYkmlpggmfsntdp----------TwVDcLYFSasThtTVGYGDL
Kcv_{PBCV1}	-------ilaMFvmiYkffpggfennfsvaNpdkka---SwIDcIYFgVTThSTVGfGDI
Kesv	--lsnlIllVVFaelYwqldqgddhthfgfs--------SaIDayYFSavTsSsVGYGDL
$Kmpv_{PL1}$	--idkfklvItvallYgylyslmepEefgfk--------TaLDpyYFSfTTmSsVGYGDf
$Kmpv_{SP1}$	--idkfkliVIvallYgfiysrmdpEefgfs--------SpLDpyYFSfTTmSsVGYGDs
Kbpv1	--nkavtiaLIitlvYgylystmk-Edfgfsd-------dpLDpyYFSLmTmSTVGYGDf
Kmpv1	--mktvLniLIittlYgllyskmkpEhfnf-K--------SpLDpfYFSfTTmSsVGYGDf

KrvSA1	vPygpiAssLnaLQmVsifiins
MthK	sPsTplgmyftVtliVlgIgtfa
Kjk1	tPqTflgKLsVIIyifagVgaVa
KcsA	yPvTlwgrLvaVVvmVagItsfg
MloK1	iPqsfagrVLagavmMsgIgifg
KirBac3.1	hPqTvyAhMvatLEifvgmsgIa
**P2Kpov**	vPiTnksrIpVMIiqsliIlrVl
**P1Kpov**	sPaTkksKIyVIIiqtfiLleIl
Kaav	tPssistrtLaMIQacstfyiLm
Kfv	yPlsnklKsLIIIQlfttItiLf
Kolpv2	yPiTnlsKILVsIQgLitLslIl
KccvKB1	fPvTytsKtLagIQgLltVslIi
Khev	yPvTlasKLLtgIQgLltVslIi
Ktv1	yPvitvsKMIaVtKmLlfLviLm
$Kotu_{6}2$	tPksniAKfLtILlqfistigIi
$Kotu_{6}1$	sPkshitKILVIMmqMfvVigfi
Kolv4	yPksstArtLVMLQqamlIvgVv
$Kotv_{RT}$	yPispmAKMIVMVQqsvlIlgIl
Kcv_{ATCV}	tPkspvAKLtatahmLivfaiVi
Kcv_{PBCV1}	lPkTtgAKLctIahiVtvffiVl
Kesv	lPkTpkAKLLtIahiLamffvml
$Kmpv_{PLs}$	sPiTgrAKMLaMtQqafifgeIl
$Kmpv_{SP1}$	sPkTdrAKLLVMtQqVfifgeIl
Kbpv1	sPkTrrAKaLVMshhtviLveLe
Kmpv1	sPkTdaAKLvVMsQhLvmIgeLa

Supplementary Figure S7: Similarity between channel pore-doamins from viruses and prokaryotes. Multiple sequence alignment of the pore domains from viral (red) and prokaryotic K^+ channels (blue). Alignment was performed with pore domains from GY/FG motif plus 29 amino acids upstream and 51 amino acids downstream of this filter motif. The alignment contains P1 and P2 from Kpov and viral channels listed in Table plus procaryotic channels KcsA, MloK1, KirBac3.1, Kjk1, and MthK (Supplementary Table 1). The eight amino acids of the filter motifs are in bold. In the alignment similar residues color-coded with average BLOSUM62 scores of *Max: 3.0*, *Mid: 1.5*, *Low: 0.5*

Supplementary Table 1

Established and putative K^+ channels from various organisms with information on Gene bank number, annotation in gene bank and number of amino acids in the protein monomer. Color coding: Proteins with a K2P type structure, of non-viral and viral origin as well as K^+ channels with a canonical 2-TMD structure of viral origin or prokaryotic origin. In case of aquatic organisms/viruses the habitat in fresh water (fw) or salt water (sw) is provided. Nomenclature of K^+ channels and putative K^+ channels is as follows: i. eukaryotic K^+ channels according to HUGO gene nomenclature committee (https://genenames.org/data/genegroup/#!/group/277) and common alias name in brackets, ii. bacterial K^+ channels according to Kuo et al., 2005 iii. putative K^+ channels from lower organisms are named here as K for K^+-channel plus abbreviation of species e.g. Khf stands for **K**$^+$ channel from ***H**ondeaa **f**ermntalgiana*. iv. For established and putative K^+ channels from viruses, the names are composed of K for K^+-channel, and the name of the virus, which encodes the sequence, e.g., Kmpv is **K**$^+$ channel from **M**. **p**usilla **v**irus; where appropriate an additional index specifies the virus isolate; $Kotv_{RT}$ is the K^+ channel from Ostreococcus tauri virus isolate RT. A K^+ function of many viral proteins has been tested either by functional reconstitution in planar lipid bilayers or by heterologous expression in mammalian cells or Xenopus oocytes. Proteins which show in these assays K^+ channel function are labeled with (+) while those which exhibited no function are indicated by (-). For proteins identified via a BLAST search in the NCBI database using the Kpov sequence as the search term, the E-value and the percentage identity relative to the search term are also given.

Channel/ protein	Organism (Phylum)	Gene bank nr	annotation	e-value Identity (%)	AA	Habi tate	Funct.
Kpov	Pyramimonas orientalis Virus (Imitervirales)	QOI90491.1	Hypoth. protein		178	sw	+
$KccKB1v_{KB1}$	Chrysochromulina sp. virus Kāne'ohe Bay 1 (*Imitervirales*)	QOC69404.1	Putative potassium channel		175	sw	-
Kfv_{SA1}	Florenciella virus Station ALOHA 1(*Imitervirales*)	QOC69405.1	Putative potassium channel		144	sw	-
Khev	Haptolina ericina virus (*Imitervirales*)	XPO57177.1	Putative ion channel domai containg protein		166	sw	-
Ktv1	Tetraselmis-Virus 1 (TetV1) (*Imitervirales*)	YP_010783067.1	Putative ion channel		81	sw	+
Krv_{SA1}	Rhizochromulina virus Station ALOHA 1 (*Imitervirales*)	QOC69403.1	Putative potassium channel		101	sw	-
Kaav	Aureococcus anaphagef-ferenses virus (*Imitervirales*)	YP_009052228.1	Putative ion channel domai containg protein		156	sw	
$Kotu_6 1$	Virus vOTU6 (*Imitervirales*)	XXH85982.1	Hypothetical protein		102	sw	
$Kotu_6 2$	Virus vOTU6 (*Imitervirales*)	XXH87252.1	Hypothetical protein		101	sw	
Kcv_{ATCV1}	Chlorovirus heliozoae (*Algalvirales*)	YP_001427066.1	Hypothetical protein		82	fw	+
Kcv_{PBCV1}	Paramecium bursaria virus-1 (*Algalvirales*)	NP_048599.1	Potassium ion channel		94	fw	+
Kesv	Ectocarpus siliculosus virus 1 (*Algalvirales*)	NP_077708.1	Hypothetical protein		124	sw	-
Kmpv1	Micromonas sp. RCC1109 virus MpV1 (*Algalvirales*)	YP_004062056.1	cAMP-dependent Kef-type K^+ transporter		79	sw	+
$Kmpv_{PL1}$	Micromonas pusilla virus PL1 (*Algalvirales*)	AET43568.1	Hypothetical protein		85	sw	+
$Kmpv_{SP1}$	Micromonas pusilla virus SP1 (*Algalvirales*)	YP_009664966.1	Hypothetical protein		86	sw	+
Kolv2	Unknown virus organic lake (*Algalvirales*)	ADX06223.1	Hypothetical protein		105	sw	-
Kolv4	Ostreococcus lucimarinus virus OlV4 (Prasinovirus)	AET84496.1	Hypothetical protein		102	sw	-

$Kotv_{RT}$	Ostreococcus tauri virus RT-2011 (Prasinovirus)	AFC34969.1	Hypothetical protein		104	sw	+
KcsA	*Streptomyces lividans* (Bacteria)	3PJS_K	Voltage gated potassium channel		166		
KirBac3.1	*Burkholderia thailandensis* (Bacteria)	ABC37093.1	Similar to potassium channel protein		532		
Kjk1	*Methanocaldococcus jannaschii* (Archaea)	WP_010869633.1	Potassium channel protein MjK1		333		
MloK1	*Mesorhizobium loti* (Bacteria)	WP_027062188.1	Cyclic nucleotide gated potassium channel		363		
MthK	*Methanothermobacter thermautotrophicus* (Archaea)	WP_010877130.1	Calcium-gated potassium channel MthK		336		
Khf	*Hondaea fermentalgiana* (Bigyra)	GBG27747.1	Two-pore potassium channel 1	5e-7 26.5%	330	sw	
Kdt	*Durusdinium trenchii* (Alveolata)	CAK9111842.1	Two-pore potassium channel	1e-6 26.5%	620	sw	
Kab	*Aphelenchoides besseyi* (Nematoda)	KAI6231427.1	Potassium channel Subfam. K member	7e-5 28.1%	757	fw	
Ksr	*Salpingoeca rosetta* (Craspedida)	XP_004991099.1	Uncharacterized protein PTSG_08078	8e-5 24.6%	1135	sw	
Kpp	*Pycnococcus provasolii (Chlorophyta)*	GHP04003.1	potassium channel	8e-4 24.0%	634	sw	
Kct	*Cymbomonas tetramitiformis (Chlorophyta)*	KAK3286188.1	Hypothetical protein CYMTET_6243	0.041 31.6%	323	sw	
Kot	*Ostreococcus tauri* (Chlorophyta)	OUS46104.1	Putative outward-rectifying potassium channel KCO1	4e-5 48.7%	402	sw	
Knf	*Naegeria fowleri* (Protist)	CAG4715687.1	Unnamed protein product		407	fw	
Kng2	*Naegeria gruberi* (Protist)	XP_002682402.1	Potassium channel		623	fw	
Kng1	*Naegeria gruberi* (Protist)	XM_002670992.1	Uncharacterized protein		546	fw	
Knl	*Naegeria lovaniensis* (Protist)	XP_044555360.1	Uncharacterized protein		613	fw	
Kfa	*Fopius arisanus* (Insect)	XM_011317030.1	Open rectifier potassium channel protein		1094		
AtTPK1 (KCO1)	*Arbidopsis thaliana* (Spermatophyta)	NP_200374.1	Outward rectifying potassium channel protein		363	fw	
KCNK (TWIK1)	*Homo sapiens* (Mamalia)	3UKM_A	TWIK-1 channel		280		

Supplementary Table 2

Primary sequence of viral $Kmpv_1$ channel was compared with sequences of six other established or putative channel proteins from different viruses using BLASTP algorithm. The similarity of the channel proteins is expressed as % identity of the local alignments and e values (e-value) relative to $Kmpv_1$. Proteins with experimentally confirmed K^+ channel activity are shown in **bold**. Experimental work with information on channel activity or failures to detect channel activity is provided in references: (1), (2), (3), (4).

Channel/protein	Annotation/ confirmed function	Virus origin	%identity/ e-value	Gene bank nr.
**$Kmpv_1$** (1)	K^+ channel	Micromonas sp. RCC1109 virus		YP_004062056.1
**$Kmpv_{SP1}$** (2)	K^+ channel	Micromonas pusilla virus SP1	66/3e-67	YP_009664966.1
**$Kmpv_{PL1}$** (2)	K^+ channel	Micromonas pusilla virus PL1	58/5e-23	ART43568.1
Kmcv	Hypothetical protein	Micromonas commando virus	49/2e-14	XCA47324.1
**$Kotv_{RT}$** (3)	K^+ channel	Ostreococcus tauri virus RT-2011	41/1e-4	AFC34969.1
$Kolv_4$ (3)	K^+ channel like protein	Ostreococcus lucimarinus virus Olv4	39/5e-4	AET84496.1
Kesv (4)	K^+ channel like protein	Ectocarpus siliculosus virus 1	42/7e-8	NP_077708.1

Supplementary Table 3: Genomes of different *Pyramimonas* species in NCBI data bank searched for protein similar to Kpov

Pyramimonas parkeae (taxid:36894)
Pyramimonas australis (taxid:132611)
Pyramimonas diskoicola (taxid:1690225)
Pyramimonas gelidicola (taxid: 471943)
Pyramimonas reticulata (taxid:51322)
Pyramimonas disomata (taxid:67781)
Pyramimonas amylifera (taxid:1486889)
Pyramimonas montana (taxid:36882)
Pyramimonas olivacea (taxid:36892)
Pyramimonas, unclassified (taxid:2302909)

References

1. F. Siotto, *et al.*, Viruses infecting marine picoplancton encode functional potassium ion channels. *Virology* **466–467**, 103–111 (2014).
2. D. Eckert, *et al.*, A small viral potassium ion channel with an inherent inward rectification. *Channels* **13**, 124–135 (2019).
3. F. Siotto, "Mining and analysis of new viral potassium channel proteins A structure and function study of new viral potassium channels from marine picoplankton and chlorella viruses," TU Darmstadt, Darmstadt. (2018).
4. A. J. Engel, *et al.*, Light-Regulated Transcription of a Mitochondrial-Targeted K+ Channel. *Cells* **9**, 2507 (2020).
